# Phytochemical characterization and anticancer potential of *Psidium cattleianum* Sabine aerial parts’ *n-*hexane extract and its subfractions

**DOI:** 10.1371/journal.pone.0335134

**Published:** 2025-10-27

**Authors:** Eman M. El-Deeb, Fatma A. Moharram, Hussein S. Taha, Mohamed R. Elgindi, Kuei-Hung Lai, Hassan Y. Ebrahim, Heba E. Elsayed

**Affiliations:** 1 Pharmacognosy Department, Faculty of Pharmacy, October 6 University, Giza, Egypt; 2 Pharmacognosy Department, Faculty of Pharmacy, Helwan University, Ein Helwan, Cairo, Egypt; 3 Department of Plant Biotechnology, Genetic Engineering and Biotechnology Division, National Research Centre, Cairo, Egypt; 4 Graduate Institute of Pharmacognosy, College of Pharmacy, Taipei Medical University, Taipei, Taiwan; 5 PhD Program in Clinical Drug Development of Herbal Medicine, College of Pharmacy, Taipei Medical University, Taipei, Taiwan; 6 Traditional Herbal Medicine Research Center, Taipei Medical University Hospital, Taipei, Taiwan; 7 Department of Biomedical Sciences, Edward Via College of Osteopathic Medicine, Monroe, Louisiana, United States of America; University of Brescia: Universita degli Studi di Brescia, ITALY

## Abstract

*Psidium cattleianum* Sabine (Family Myrtaceae) is a Brazilian native shrub, valued for its diverse health and therapeutic attributes. The current study investigated the phytochemical profile along with the anticancer activities of the *n-*hexane extract (HE) of *P. cattleianum* aerial parts and its subfractions. GC-MS and HPTLC-MS were used for phytochemical analysis. The human breast adenocarcinoma cells (MCF-7) and the human colon cancer cells (HCT-116) were used to investigate the anticancer effect in the viability, migration, and clonogenic assays. The GC-MS analysis of HE identified thirty-two components categorized mainly into terpenes, hydrocarbons, and sterols. *β*-caryophyllene oxide (12.07%) and humulene (7.42%) were the most abundant oxygenated and non-oxygenated metabolites, respectively. Concerning HE’s subfractions, fraction I is prolific with caryophyllene oxide (19.48%) and humulene (9.96%), while fraction II was rich in caryophyllene oxide (6.89%). HPTLC-MS analysis of fractions III-V identified the presence of nonadecatetraene, heptacosanol, and dihydroxy-oxo-ursenoic acid in fraction III; caryophyllene and littordial C in fraction IV, while guavanoic acid, *p*-coumaroyl caffeoylquinic acid, cholestane heptol, tocopherol, heptacosanedione, and *trans*-calamenene in fraction V. Concerning the anticancer results, the HE showed potent cytotoxicity with IC_50_ 29.18 ± 0.43 μg/mL (MCF-7) and 56.55 ± 6.8 μg/mL (HCT-116). In addition, at maximum tested doses approximating ½ IC_50_ (15 and 28 μg/mL) in cytotoxicity assay, it displayed significant percent wound closure of 22.78 ± 2.13% and 12.76 ± 1.88%, respectively. While at doses corresponding to ¼ IC_50_ (7.5 and 14 μg/mL), the HE displayed a colony formation efficiency of 2% and 0% on MCF-7 and HCT-116, respectively. Subfractions I and II, rich in caryophyllane sesquiterpenes, such as caryophyllene oxide, showed the best activity in all assays. Molecular docking of *β*-caryophyllene oxide, as the most identified bioactive metabolite, revealed an energetically favorable binding pose driven through hydrophobic interactions at the estrogen receptor ligand binding domain. The study endorses *P. cattleianum* HE and its selected fractions in the control of breast and colon cancers; however, further investigation into an appropriate *in vivo* model is required.

## Introduction

Plants are exclusive sources of natural products (NPs) that possess unique chemical scaffolds and hold promising therapeutic potential [1]. Nowadays, NPs have global appraisal as an indispensable source of lead compounds for drug discovery [2]. However, despite the ubiquity of plants, the scientific community has expanded the scope to investigate promising species, aiming to develop and design new NP-based therapeutics [1,2]. *Psidium cattleianum* Sabine (Family Myrtaceae), commonly recognized as strawberry guava, or Araçá is a Brazilian marketable fruit native to the Amazonian Basin [3]. The species is appreciated for its aesthetic appeal, edible fruit, and therapeutic properties, which include antimicrobial, anti-inflammatory, antioxidant, antidiabetic, and anticancer effects [4–6]. The anticancer potential of *P. cattleianum* has been addressed in limited reports. For instance, the fruit’s acetone and aqueous extracts showed an antiproliferative impact on MCF-7 and Caco-2 human cancer cells [6]. The essential oil from leaves displayed cytotoxicity to MCF-7 cells [7], while the methanol extract of the aerial parts moderately affects the viability of MCF-7, HepG2, and K-562 cell lines [8]. The pharmacological activities and health benefits mentioned earlier are attributed to diverse phytoconstituents, including vitamin C, polyphenols, carotenoids, terpenes, and essential oils [4,5]. Although the available literature underscores the phytochemical richness of *P. cattleianum* Sabine, the species still merits further investigation.

The structure-complexity of NPs presents significant challenges, especially in terms of isolation, identification, and screening [1]. Recently, these challenges have been addressed by various scientific and industrial advancements. Such as adopting innovative analytical techniques and applying state-of-the-art screening approaches [9–11]. Among these, Gas Chromatography-Mass Spectrometry (GC–MS) is regarded as a gold standard analytical technique [9]. In this technique, volatile NPs are separated based on the solvation capability of gas chromatography, while taking advantage of the multidimensional detection by mass spectrometry, even at low concentration [9]. Another useful analytical technique that has gained prominence is high-performance thin-layer chromatography hyphenated to mass spectrometry (HPTLC-MS) [10]. HPTLC is a rapid, inexpensive, and versatile chromatographic tool, ideal for separating complicated extracts with high sensitivity and reproducibility [10]. It utilizes customized plates and instrumental resources for sample application and detection [11]. The hyphenation of HPTLC to MS offers a great advantage in that the questioned bands are assigned to the MS, followed by sensitive mass spectrometric data [11]. Therefore, using GC-MS and HPTLC-MS for the detection and identification of NPs in an extract or a fraction is an effective strategy.

Cancer is a generic term coined for a heterogeneous disease whose cells have the swift capability to multiply, invade, and metastasize beyond their typical frontiers, causing fatality [12]. An updated estimate for the global burden of cancer incidence and mortality rates, states that breast cancer (BC) is the commonly diagnosed cancer in females worldwide, with an incidence rate of 11.6% [1]. In terms of mortality rate, BC is the leading cause of cancer fatalities in developed countries, constituting 6.9% [12,13]. On the other hand, colorectal cancer (CRC) is the second leading cause of cancer-related deaths and the third most common malignancy globally. It accounts for 9.6% of new cancer cases and 9.3% of cancer fatalities annually [12,13]. Despite the significant burden imposed by BC and CRC, current pharmacotherapeutics often carry limitations such as intolerability and acquired resistance, highlighting the dire need for safe and effective complementary approaches [14]. While natural products have been increasingly investigated in oncological research, the phytochemical spectrum and anticancer characteristics of some species, such as *Psidium cattleianum* Sabine, have not been sufficiently addressed. Herein, we report the first comprehensive phytochemical profiling of *P. cattleianum* Sabine *n*-hexane extract (HE) and its sub-fractions using GC-MS and HPTLC-MS. Additionally, we investigate the *in vitro* anticancer potential in in-house available breast cancer (BC) and colorectal cancer (CRC) cell lines. Ultimately, we correlate the cellular effects to molecular targets using computational modeling.

## Materials and methods

### General experimental materials

*n*-Hexane, methanol (MeOH), dichloromethane (DCM), sulphuric acid (H_2_SO_4_), and ethyl acetate (EtOAc) were obtained from El-Nasr Pharmaceutical Chemicals Company (Cairo, Egypt), while *p*-anisaldehyde was purchased from Sigma-Aldrich Inc. (St. Louis, MO, United States). Silica gel 60 and silica gel plates (Sigma-Aldrich Inc., St. Louis, MO, United States) were used for column chromatography (CC) and thin layer chromatography (TLC), respectively.

### Plant material

Fresh aerial parts of *Psidium cattleianum* Sabine were collected in March 2021 from Mazhar Botanical Garden (Cairo, Egypt). The species was collected according to the local garden guidelines with prior permission from the garden authorities. The taxonomic identification was performed at Mazhar Botanical Garden, Cairo, Egypt, by Dr. Trease Labib. A voucher was registered under 01Pca/2021 and deposited at the Herbarium of the Faculty of Pharmacy, Helwan University, Cairo, Egypt.

### Preparation and fractionation of the *n*-hexane extract (HE)

Fresh aerial parts (2.75 Kg) were dried in the shade and coarsely ground to afford 1.45 Kg of dry powder. The powder was exhaustedly extracted with 80% aq. methanol (3 × 5 L) under reflux, followed by evaporating the solvent under a vacuum at low temperature to yield 380 g dry residue. Subsequently, the residue was extracted with *n*-hexane under reflux (3 x 1 L) followed by solvent evaporation to afford 42 g and 315 g of *n-*hexane extract (HE) and aqueous methanol residue, respectively. A small-scale HE (1 g) was subjected to GC-MS analysis, while 5 g was held for the biological assessment. On the other side, the remaining HE (33 g) was fractionated on silica gel 60 using vacuum liquid chromatography (VLC). The VLC was eluted using stepwise gradients, starting with 100% *n*-hexane, followed by increasing concentrations of DCM and then EtOAc up to 100%. Eluted fractions were examined for their composition on silica gel G60 TLC, followed by visualization under UV light, and sprayed with *p*-anisaldehyde/H_2_SO_4_ reagent. Seven collective fractions (Fr) were recognized and labeled as Fr I–VII. Collective Fr I (1.5 mL) and Fr II (0.5 mL) were eluted with 100% *n*-hexane and analysed using GC-MS. Collective Fr III (8 g, *n*-hexane: DCM 50:50), Fr IV (6.4 g, 100% DCM), Fr V (4.2 g, DCM: EtOAc 80:20), Fr VI (3 g, DCM: EtOAc 50:50), and Fr VII (6.5 g, DCM: EtOAc, 30:60) were investigated using HPTLC-MS.

### Gas Chromatography-Mass Spectrometry (GC-MS) parameters and conditions for the analysis of the HE, collective fractions I and II

It was implemented on Shimadzu GC-MS-QP2010 (Tokyo, Japan) using a split-splitless injector with Rtx-1MS Column (Restek, PA, United States). The column’s initial temperature was set at 45 °C for 2 min (isothermal), then it was programmed to 300 °C at a rate of 5 °C/min and remained constant at 300 °C for 5 min (isothermal). The flame ionization temperature is set at 280 °C. The carrier gas (Helium) is used with a flow rate of 1.41 mL/min. The interface and ion source temperatures for mass analysis are 280 °C and 200 °C, respectively, and the electron ionization voltage was 70 eV (scanning range is 35–500 amu). The sample (1 μL) was injected by split mode (1:15), and the separated compounds were identified by comparing their Kovats’ retention indices (RI) with those of the standard *n*-alkanes (C_8_-C_28_), also, matching their MS spectra with those mentioned in the NIST and Wiley database (similarity index > 90%) [15].

### High-Performance Thin Layer Chromatography-Mass Spectrometry (HPTLC-MS) analysis for collective fractions III-V

The analysis was performed using an LC-MS/MS system (Nexera with LCMS-8045, Shimadzu Corporation, Kyoto, Japan) – HPLC (Nexera LC-30AD) equipped with a photodiode array detector (LC-2030/2040) with detection wavelengths of 235, 254, and 280 nm with λ_max_ absorption at 220–400 nm and coupled to triple quadrupole mass spectrometer (Nexera with LCMS-8045, Shimadzu Corporation, Kyoto, Japan). The HPTLC plate was applied to the CAMAG-TLC interfaced to the LC-MS system. Isocratic elution was performed using 70% MeOH. Positive and negative modes were operated during the LC-MS/MS analysis with an electrospray ionization (ESI) interface. The acquisition mode was set to Q3 scan. The scan speed was 909 U/sec with *m/z* range 100–1000. The event time was one second, and the interface voltage was set at 4 KV. The interface temperature was 300 °C, while the desolvation temperature was 526 °C. Flow rates were adjusted as follows: drying gas (10 L/min), nebulizing gas (3 L/min), and heating gas (10 L/min). Heat block temperature was 400 °C. LC-MS/MS data were collected and processed by Lab Solutions software (Shimadzu, Kyoto, Japan).

### Anticancer activity

#### Cell lines and culture conditions.

The MCF-7 human breast adenocarcinoma and the HCT-116 human colon cancer cells were supplied by the Egyptian Holding Company for Biological Products and Vaccines (VACSERA, Giza, Egypt). The cells were cultured in Roswell Park Memorial Institute (RPMI))-1640 media (Lonza biosciences, Verviers, Belgium) supplemented with 2% fetal bovine serum (FBS) (Serana Europe GmbH, Pessin, Germany), 1% antibiotic/antimycotic solution (Sigma Ald., St. Louis, Mo., United States), and incubated at 37 °C with 5% CO_2_ humidified atmosphere. Sterile treated culture plates were purchased from the NEST Biotechnology Co., Ltd. (Wuxi, China). 3-(4,5-dimethylthiazolyl2)-2,5-diphenyltetrazolium bromide (MTT) was obtained from Solarbio Science & Technology Co., Ltd. (Beijing, China).

#### Stock solutions.

The HE and the collective fractions I-V were prepared in sterile, biological-grade DMSO (Sigma-Aldrich Inc., St. Louis, MO, United States) as a stock solution with a final concentration set up to 1 mg/10 μL. Each stock solution was subjected to a two-fold serial dilution using RPMI media supplemented with 2% FBS to prepare the intended final treatment concentrations for the viability assay, while prepared as planned in the wound-healing and clonogenic assays.

#### Cell-viability assay.

MCF-7 and HCT-116 cells were loaded separately in a 96-well culture plate at a density of 1 × 10^5^ cells/100 µL/well. Plated cells were incubated until they developed a confluent monolayer. Thereafter, media were removed, and cells were treated with different concentrations of HE or its sub-fractions I-V (1000–7.81 μg/mL) and vehicle (as a negative control). All plates were re-incubated at 37 °C and 5% CO_2_ humidified atmosphere for 24 h, after which media were replaced with 100 μL RPMI-1640 containing MTT (0.5 mg/mL). The plates were re-incubated at 37 °C till the formation of insoluble formazan crystals. Subsequently, the crystals were solubilized by adding 100 μL DMSO, and the absorbance was measured at 560 nm using a microplate reader (Mindray Bio-Medical Electronics Co., Ltd., Shenzhen, China) [16,17].

#### Migration assay.

In short, cancer cells were cultured in 6-well treated culture plates and incubated overnight or until the cells reached confluency. Subsequently, a wound was created using a 200 μL pipette tip at an angle. Cell debris was washed with PBS, and wounds were instantly imaged (W_0_). Subsequently, plated cells received the tested samples at two concentrations, equivalent to ½ IC_50_ and ¼ IC_50_ in the viability assay. Samples were prepared in RPMI-1640 media containing 1% FBS, while control wells received the vehicle. Plates were re-incubated until the wound was nearly closed. Next, the media were removed, and the wells were washed, fixed with 10% formalin, and imaged using a stereo microscope (ACCU-SCOPE Inc., NY, United States) [18].


$$\%\;\text{Wound}\;\text{closure}\;=\;\frac{{\text{Area}\;\text{of}\;\text{the}\;\text{wound}}_{\text{Initial}}-{\text{Area}\;\text{of}\;\text{the}\;\text{wound}}_{\text{Final}}}{{\text{Area}\;\text{of}\;\text{the}\;\text{wound}\;}_{\text{Initial}}}\;\times \text{1}\text{0}\text{0}$$

#### Colony-forming efficiency assay.

Briefly, cells were seeded in culture plates at a density of 1 × 10^5^ cells in RPMI-1640 medium supplemented with 2% FBS and incubated at 37 °C until cells were attached. Subsequently, media were gently removed, cells were washed with phosphate-buffer saline (PBS), and then treated with HE or selected fractions for 24 h at a concentration equivalent to their ¼ IC_50_ in the viability assay. By the end of the treatment period, the media were aspirated. Surviving cells were counted and seeded in a 6-well-treated culture plate (NEST Biotechnology Co., Ltd, Wuxi, China) at a density of 100 cells/mL/well in RPMI-1640 medium supplemented with 2% FBS and incubated at 37 °C for a time that is equivalent to at least six potential cell divisions (2–3 weeks). Formed colonies were washed, fixed with 10% formalin, stained with 0.5%w/v crystal violet (Sigma-Aldrich Inc., St. Louis, MO, United States), and counted using a stereo microscope (ACCU-SCOPE Inc., NY, United States) [19]. Colony-forming efficiency (CFE), also referred to as plating efficiency (PE), is the ability of a single cell to form a colony and is calculated as follows:


$$\text{Colony}\text{-}\text{forming}\;\text{efficiency}\;(\text{CFE})\;\text{or}\;\text{plating}\;\text{efficiency}\;(\text{PE})\;=\;\frac{\text{number\textasciitilde{}of}\;\text{colonies}\;\text{formed}}{\text{number}\;\text{of}\;\text{plated}\;\text{cells}\;(\text{100}\;\text{c}ell)}\;\text{X}\;\text{1}\text{0}\text{0}$$

$$\text{Surviving}\;\text{fraction}\;(\text{SF})\;=\frac{\left(\frac{\text{No}.\;\text{of}\;\text{colonies}}{\text{No}.\;\text{of}\;\text{plated}\;\text{cells}}\right)}{\text{Plating}\;\text{efficiency}\;\text{of}\;\text{control}}\times \text{100}$$

### Statistical analysis

All experiments were performed in triplicate in three independent investigations (n = 3). Data analysis was performed using GraphPad Prism Software version 5.0 (La Jolla, CA, United States) and represented as mean ± SD or mean ± SEM as stated in each experiment. Values were compared using the student’s *t*-test, with a *p-value* <0.05 considered statistically significant relative to control or *n*-hexane extract and denoted with an asterisk.

### Molecular docking

The *in-silico* experiments were executed using AutoDock Vina v1.2.x [20,21] installed on a SAMSUNG workstation with Intel(R) Core (TM) i7–6500U CPU @ 2.5 GHz processor and 12.0 GB RAM. The 2.8 Å X-ray crystal structure of human estrogen receptor alpha ligand binding domain complexed with its cognate ligand estradiol (hERαLBD) was retrieved from the Protein Data Bank (PDB) entry 1A52 [22,23]. 1A52 PDB file was imported into the AutoDock Vina interface, where polar hydrogens and partial charges were added, water molecules and ions were removed, and then the PDPQT grid dimension file was exported. A three-dimensional docking grid box was then generated and centered around estradiol, with a size of 28 points in X, Y, and Z coordinates. The 2D structure of *β*-caryophyllene oxide was sketched using the ChemDraw User Interface version 15.0, and the 3D structure was created using Chem3D and then saved as a PDB file. The 3D *β*-caryophyllene oxide structure was imported to the AutoDock Vina v1.2.x interface, the torsion tree was built to identify rotatable, nonrotatable, and unrottable bonds, and finally exported as a PDPQT file. The empirical docking scoring function combined various energy terms, including hydrogen bonding, hydrophobic interactions, desolvation, and conformational entropy penalties of ligand-protein molecular interactions. The binding poses of the most energetically favorable binding interactions determined by the lowest predicted binding free energy were implemented to rank the different ligand binding poses, with a maximum of 10 poses.

## Results

### Phytochemical profile of the *n*-hexane extract and its sub-fractions

Herein, the *n*-hexane extract (HE) of *P. cattleianum* Sabine aerial parts was prepared as stated in the Materials and Methods section. The HE was subjected to GC-MS analysis to elucidate its chemical profile for the first time. Table 1 outlines the exclusive identification of thirty-two metabolites, which represent 79.12% of the total detected peaks (S1 Fig.). The identified compounds belonged to various classes, including sesquiterpenes, diterpenes, triterpenes, hydrocarbons, and sterols. Oxygenated compounds constituted approximately 51.04% among which caryophyllene oxide (12.07%), tocopherol (7.34%), *α-*amyrins (6.31%), and humulenol-II (4.06%) were the major identified constituents. On the other hand, non-oxygenated components comprised 28.08%, and are predominated by humulene (7.42%), squalene (3.51%), and 3,5,11-eudesmatriene (2.41%)

**Table 1:** Identified constituents from *P. cattleianum* aerial parts’ *n*-hexane extract (HE) by using GC-MS analysis.

No.	RI_exp_	RI_ref_	Identified components	MF	%Area
1	1344	1344	*α*-cubebene	C_15_H_24_	1.17
2	1386	1411	alloaromadendrene	C_15_H_24_	2.08
3	1412	1428	*β*-copaene	C_15_H_24_	0.34
4	1430	1430	*cis*, *α*-bergamotene	C_15_H_24_	0.31
5	1431	1440	*γ*-elemene	C_15_H_24_	1.39
6	1435	1432	*cis*-muurola-4(15),5-diene	C_15_H_24_	1.12
7	1436	1436	humulene	C_15_H_24_	7.42
8	1444	1470	*γ-c*adinene	C_15_H_24_	0.61
9	1469	1469	eudesma-4(14),11-diene	C_15_H_24_	0.30
10	1469	1472	cadina-3,9-diene	C_15_H_24_	1.10
11	1477	1477	*β*-chamigrene	C_15_H_24_	0.64
12	1480	1480	*α-*muurolene	C_15_H_24_	0.30
13	1484	1494	3,5,11-eudesmatriene	C_15_H_22_	2.41
14	1515	1515	germacrene D	C_15_H_24_	0.66
15	1523	1522	*β*-guaiene	C_15_H_24_	1.42
16	1537	1532	calamenene	C_15_H_22_	0.19
17	1561	1561	caryophyllene oxide	C_15_H_24_O	12.07
18	1564	1564	nerolidol	C_15_H_26_O	1.97
19	1580	1605	*tau*-cadinol	C_15_H_26_O	1.33
20	1580	1580	(-)-globulol	C_15_H_26_O	2.26
21	1602	1632	humulenol-II	C_15_H_24_O	4.06
22	1719	1710	*β*-bisabolol	C_15_H_26_O	0.29
23	1774	1790	neophytadiene	C_20_H_38_	2.14
24	1800	1808	hexadecanal	C_16_H_32_O	3.01
25	2087	2087	phytol	C_20_H_40_O	0.50
26	2731	3187	*β*-sitosterol	C_29_H_50_O	4.33
27	2794	2814	squalene	C_30_H_50_	3.51
28	2836	2860.5	*α*-tocospiro A	C_29_H_50_O_4_	3.26
29	2871	2718.6	stigmasta-3,5-diene	C_29_H_48_	0.97
30	3149	3149.5	tocopherol	C_29_H_50_O_2_	7.34
31	3324	3337	*β*-amyrin	C_30_H_50_O	4.31
32	3380	3376	*α*-amyrin	C_30_H_50_O	6.31
			**Total identified components**		79.12
			**Oxygenated compounds**		51.04
			Sesquiterpenes		21.98
			Triterpenes		10.62
			Others		18.44
			**Non-oxygenated compounds**		28.08
			Sesquiterpenes		21.46
			Triterpenes		3.51
			Others		3.11

**RI**_**exp**_, experimental retention index**; RI**_**ref**_**.,** reference retention index; **MF**: molecular formula.

Consequently, the HE was fractionated using VLC, affording seven collective fractions I-VII. Fractions I and II are oily in consistency and possess an aromatic odor; hence, we recommend profiling their chemical composition using GC-MS. The analysis results of fraction I (Table 2, S1 Fig.) identified twenty compounds representing 78.03% of the detected peaks. In contrast to the HE, fraction I was copious with non-oxygenated sesquiterpenes constituting 50.95%, followed by oxygenated sesquiterpenes (24.92%). Humulene (9.96%) and calamenene (8.23%) were the major tracked non-oxygenated sesquiterpenes, whereas caryophyllene oxide (19.48%) represented the major oxygenated component. Concerning fraction II, seventeen compounds were identified (Table 3, S1 Fig.), constituting 58.19% of the detected peaks. The tentatively identified compounds were categorized as oxygenated (37.67%) and non-oxygenated (20.52%). Caryophyllene oxide (6.89%), clovandiol (8.20%), and globulol (6.42%) were among the predominant oxygenated sesquiterpenes, while cadalene (8.62%) was among the major detected non-oxygenated.

**Table 2:** Identified constituents from fraction I of *P. cattleianum* aerial parts’ *n*-hexane extract (HE) by using GC-MS analysis.

No.	RI_exp_	RI_ref_	Compound name	MF	%Area
1	1381	1381	copaene	C_15_H_24_	3.17
2	1389	1389	*β*-bourbonene	C_15_H_24_	0.16
3	1395	1395	*β*-elemene	C_15_H_24_	0.40
4	1409	1391	7-epi-sesquithujene	C_15_H_24_	0.43
5	1427	1411	isocaryophllene	C_15_H_24_	8.95
6	1446	1441	aromandendrene	C_15_H_24_	3.62
7	1463	1463	humulene	C_15_H_24_	9.96
8	1494	1494	eudesma-4(14),11-diene	C_15_H_24_	7.37
9	1502	1501	*α*-selinene	C_15_H_24_	2.72
10	1530	1531	calamenene	C_15_H_22_	8.23
11	1544	1544	selina-4(15),7(11)-diene	C_15_H_24_	2.93
12	1551	1551	selina-3,7(11)-diene	C_15_H_24_	2.31
13	1561	1561	caryophyllene oxide	C_15_H_24_O	19.48
14	1668	1667	calamenen-10-ol	C_1__5_H_22_O	0.97
15	1684	1684	cadalene	C_15_H_18_	2.38
16	1689	1687	mustakone	C_15_H_22_O	0.70
17	1697	1697	*α*-bisabolol	C_15_H_26_O	0.50
18	1712	1707	10-*nor-*calamenen-10-one	C_14_H_18_O	0.46
19	1721	1694	(1R,7S)-germacra-4(15),5,10(14)-trien-1-*α*-ol	C_15_H_24_O	1.59
20	2834	2833	squalene	C_30_H_50_	1.70
			**Total identified components**		78.03
			**Oxygenated compounds**		25.38
			Monoterpenes		0.46
			Sesquiterpenes		24.92
			**Non-oxygenated compounds**		52.65
			Triterpenes		1.70
			Sesquiterpenes		50.95

**RI**_**exp**_, experimental retention index**; RI**_**ref**_**.,** reference retention index; **MF**: molecular formula.

**Table 3:** Identified constituents from fraction II of *P. cattleianum* aerial parts’ *n*-hexane extract (HE) by using GC-MS analysis.

No.	RI_exp_	RI_ref_	Compound name	MF	%Area
1	1393	1391	*α*-ionone	C_13_H_20_O	0.54
2	1431	1431	***γ***-elemene	C_15_H_24_	0.50
3	1458	1445	*α*-curcumene	C_15_H_22_	0.86
4	1467	1496	actinidiolide	C_11_H_16_O_2_	2.80
5	1488	1475	*α*-acoradiene	C_15_H_24_	1.50
6	1512	1513	*α*-calacorene	C_15_H_20_	0.95
7	1552	1552	caryophyllene oxide	C_15_H_24_O	6.89
8	1576	1476	valencene	C_15_H_24_	4.91
9	1592	1680	*β*-santalol	C_15_H_24_O	0.55
10	1599	1680	humulenol-II	C_15_H_24_O	5.31
11	1602	1644	caryophylla-4(12),8(13)-dien-5- *β* -ol	C_15_H_24_	3.18
12	1623	1673	allohimachalol	C_15_H_26_O	4.46
13	1632	1599	globulol	C_15_H_26_O	6.42
14	1637	1637	cadalene	C_15_H_18_	8.62
15	1739	1640	epicubenol	C_15_H_26_O	1.62
16	1819	1885	clovandiol	C_15_H_26_O_2_	8.20
17	1876	1611	*β*-oplopenone	C_15_H_24_O	0.88
			**Total identified compounds**		58.19
			**Oxygenated compounds**		37.67
			Sesquiterpenes		34.33
			Monoterpenes		3.34
			**Non-oxygenated compounds**		20.52
			Sesquiterpenes		20.52

**RI**_**exp**_, experimental retention index**; RI**_**ref**_**.,** reference retention index; **MF**: molecular formula.

Fractions III-VII were subjected to HPTLC-MS to elucidate their chemical profile. The HPTLC-MS chromatogram of fraction III revealed the presence of four major molecular ion peaks in different ionization modes (S1 Fig.). The molecular ion peak at *m/z* 261.25 in the positive mode corresponding to the molecular formula (MF) C_19_H_32_ was tentatively identified as the hydrocarbon nonadecatetraene [24]. The molecular ion peak at *m/z* 301.10 [M+H]^+^ corresponding to the MF C_18_H_36_O_3_ was elucidated as the hydroxyoctadecanoic acid [25]. Additionally, the molecular ion peak at *m/z* 397.45 [M+H] ^+^ was correlated to the fatty alcohol heptacosanol (MF C_27_H_56_O) [26], and the molecular ion peak in the negative mode at *m/z* 485.34 (MF C_30_H_46_O_5_) was ascribable to the triterpenoid dihydroxy-oxo-ursenoic acid [27]. On the other side, the interpretation of the HPTLC-MS chromatogram of fraction IV showed two major compounds that were tentatively identified as the bicyclic sesquiterpene caryophyllene (*m/z* at 205.25 [M+H] ^+^, MF C_15_H_24_) [28] and the meroterpenoid littordial C (*m/z* at 467.35 [M-H]^-^, MF C_29_H_40_O_5_) [29] (S1 Fig.). It is worth noticing that caryophyllene was detected earlier by GC-MS in the essential oil of *P. cattleianum* leaves [7] and littordial C was previously isolated from the extract of *P. cattleianum* leaves [29]. HPTLC-MS of fraction V, exhibited six major molecular ion peaks (S1 Fig.) displayed at *m/z* 501.30 [M-H]^-^, 499.30 [M-H]^-^, 483.35 [M-H]^-^, 415.30 [M-H]^-^, 409.40 [M+H]^+^, and 203.25 [M+H]^+^ ascribable to guavanoic acid (MF C_30_H_46_O_6_), *p*-coumaroyl caffeoylquinic acid (MF C_25_H_24_O_11_), cholestane heptol (MF C_27_H_48_O_7_), *β*-tochopherol (MF C_29_H_50_O_2_), heptacosanedione (MF C_27_H_52_O_2_), and calamenene (MF C_15_H_22_), respectively. Indeed, guavanoic acid, *p*-coumaroyl caffeoyl quinic acid, cholestane heptol, and heptacosanedione were detected in *P. cattleianum* extract for the first time. However, tocopherol and calamenene were previously identified in the essential oils of *P. guajava* and *P. cattleianum* aerial parts by GC-MS [7,30]. Ultimately, the phytochemical analysis of the last two collective fractions (VI and VII) displayed no definite compounds; therefore, they were not considered for further biological screening.

### Anti-cancer activity of the *n*-hexane extract (HE) and its fractions

#### Effect of HE and its sub-fractions on the viability of MCF-7 and HCT-116 cells.

**Fig 1:**
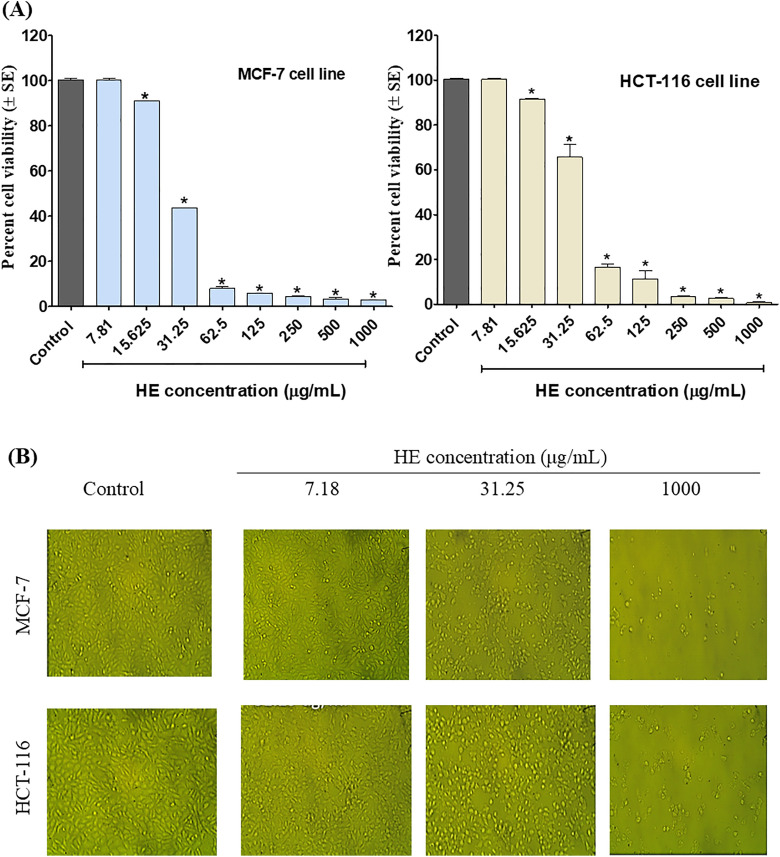
Effect of *P. cattleianum* HE on the viability of MCF-7 breast cancer and HCT-116 colorectal cancer cells in MTT assay. (A) Dose-response curve showing percent cell viability versus extract concentration (μg/mL), (B) Representative images of the treated cancer cells by HE compared to control.

The HE and its chemically noteworthy fractions have been screened for their anticancer potential against two selected cancer cell lines, *viz,* the MCF-7 and HCT-116. The results (Fig 1) showed that HE significantly reduced the viability of MCF-7 and HCT-116 cells in a dose-dependent manner with IC_50_ values of 29.18 ± 0.43 μg/mL and 56.55 ± 6.8 μg/mL, respectively.

Subsequently, fractions I-V were screened for their inhibitory potential on the viability of MCF-7 and HCT-116 cells. The results (Table 4) showed that among all tested fractions, fraction I exhibited significant (*P*<0.05) better viability inhibition against MCF-7 cells (by half-fold) than the HE. At the same time, its cytotoxicity to HCT-116 was 4.5-fold more potent than HE (*P*<0.001). We also observed a significant (*P*<0.01) reduction in the potency of fraction II on MCF (IC_50_ 49.59 ± 2.56 μg/mL), while its activity on HCT-116 was significantly 7-fold better than HE (*P*<0.01). Fraction III showed faraway viability inhibition to MCF-7, while fraction IV conserved its cytotoxicity potential (IC_50_ 52.72 ± 4.63 μg/mL) on the same cell line. Conversely, fractions III and IV showed comparable activity to HE on HCT-116 with IC_50_ values of 57.53 ± 1.01 μg/mL and 55.11 ± 6.37 μg/mL, respectively. Lastly, fraction V showed the least activity with an IC_50_ of 93.14 ± 11.7 μg/mL (HCT-116) and 225.43 ± 6.90 μg/mL (MCF-7). It is obvious from the viability assay that fraction I displayed the most potent effect, among other fractions, to both cell lines, followed by fraction II (on HCT-116).

**Table 4:** The IC_50_ (μg/mL) values of *P. cattleianum* aerial parts’ *n*-hexane extract (HE) and its collective sub-fractions I-V in MTT viability assay.

Tested samples	IC_50_ (μg/mL)	
	MCF-7	HCT-116
HE	29.18 ± 0.43	56.55 ± 6.80
Fraction I	20.64 ± 1.52*	12.15 ± 0.80***
Fraction II	49.59 ± 2.56**	15.39 ± 2.11**
Fraction III	151.50 ± 9.51****	57.53 ± 1.01 ^ns^
Fraction IV	52.72 ± 4.63**	55.11 ± 6.37 ^ns^
Fraction V	225.43 ± 6.90****	93.14 ± 11.73**

Data presented as mean ± SD (n = 3); values were compared using the student’s *t*-test and the statistical significance was denoted with an asterisk as follows: *: p < 0.05; **: p < 0.01; ***: p < 0.001; ***: p < 0.0001 vs HE; ns: statistically non-significant (p > 0.05) vs HE

#### Effect of HE and its fractions on the migration of MCF-7 and HCT-116 cells.

**Fig 2:**
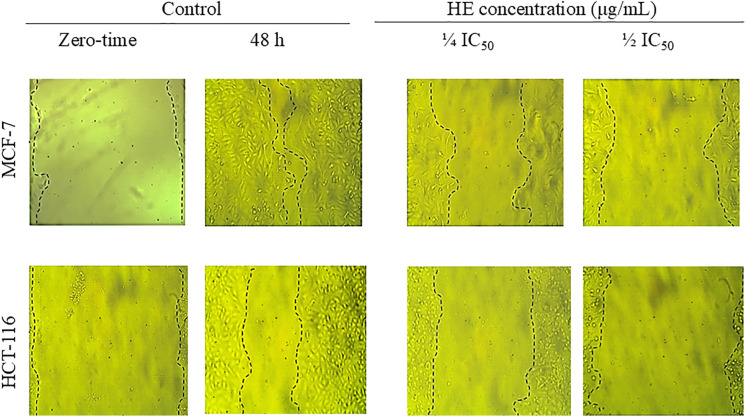
Effect of two selected doses of HE (½ IC_50_ and ¼ IC_50_ in the viability assay) on the motility of MCF-7 breast cancer and HCT-116 colorectal cancer cells in the migration assay. The figure showed representative images of the created wound in both cell lines at 0 time and 48 h post-treatment compared to vehicle control.

The results of the wound-healing assay obtained 48 h post-scratching (Fig 2, Table 5) revealed that HE possessed a significant (*P* < 0.0001), selective high inhibitory effect on the motility of HCT-116 cells, deduced from percent wound closure (12.76 ± 1.88%), at the maximum tested dose (½ IC_50_), compared to the MCF-7 (22.78 ± 2.13%). On the other hand, the extract displayed significant (P < 0.01), comparable percent wound closure on the two cell lines at the minimum tested dose (¼ IC_50_), calculated as 41.06 ± 3.60% (HCT-116) and 47.60 ± 3.53% (MCF-7) compared to vehicle control (Table 5). Subsequently, fractions I, II, and IV were screened on MCF-7, while all fractions were screened on HCT-116 (Table 5). As a general observation, the tested fractions followed the selectivity theme of the HE to the HCT-116 cell line at ½ IC_50_. Fraction II displayed significantly (*P* < 0.0001) greater inhibitory effect than HE on the mobility of HCT-116 cells, with a calculated wound closure percentage of 11.50 ± 0.75%. Conversely, fraction I displayed a superior inhibitory effect than the HE on the mobility of MCF-7 cells with a calculated wound closure percent of 17.10 ± 1.40%. Concerning the activity at the minimum tested dose, fraction II still exhibits potent wound closure inhibition for HCT-116 cells (19.98 ± 0.95%), while fraction I displayed the best effect on MCF-7 cells (39.20 ± 2.68%).

**Table 5:** Percent wound closure of *P. cattleianum* aerial parts’ *n*-hexane extract (HE) and its collective sub-fractions in wound healing assay at two screening doses (¼ IC_50_ and ½ IC_50_ in MTT assay).

Cell line	MCF-7	MCF-7	HCT-116	HCT-116
Tested Conc.	**¼ IC**_**50**_	**½ IC**_**50**_	**¼ IC**_**50**_	**½ IC**_**50**_
Control	89.67 ± 4.67	89.67 ± 4.67	65.02 ± 3.62	65.02 ± 3.62
HE	47.60 ± 3.53***	22.78 ± 2.13****	41.06 ± 3.60**	12.76 ± 1.88****
Fraction I	39.20 ± 2.68***	17.10 ± 1.40****	48.13 ± 3.29**	23.25 ± 1.92****
Fraction II	46.43 ± 2.76***	29.29 ± 3.66****	19.98 ± 0.95****	11.50 ± 0.75****
Fraction III	NT	NT	44.67 ± 2.41**	26.11 ± 1.69****
Fraction IV	58.98 ± 4.36**	31.79 ± 1.78****	39.46 ± 1.51***	21.38 ± 1.44****
Fraction V	NT	NT	29.59 ± 3.74***	26.26 ± 1.99****

NT: not tested; Data presented as mean ± SD (n = 3); values were compared using the student’s *t*-test and the statistical significance was denoted with an asterisk as follows *: p < 0.05; **: p < 0.01, ***: p < 0.001, ****: p < 0.0001 vs control

#### Effect of HE and its fractions on MCF-7 and HCT-116 colony formation.

**Fig 3:**
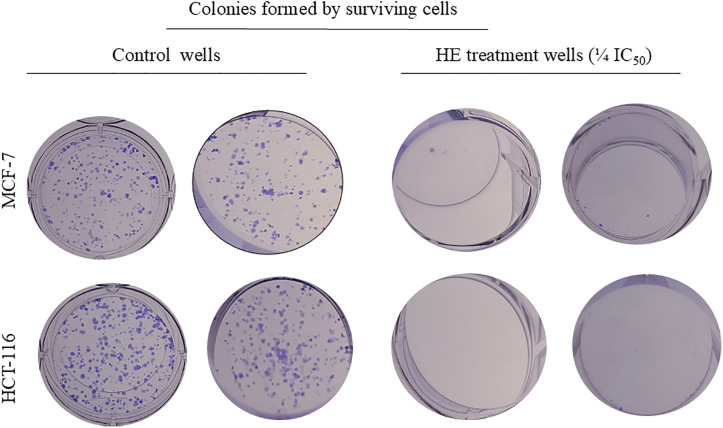
Effect of a screening dose of HE (¼ IC_50_ in the viability assay) on the colonization ability of pretreated MCF-7 breast cancer and HCT-116 colorectal cancer cells in colony formation. The figure showed representative images of the ability of the surviving cells to form colonies in the treatment group compared to the vehicle control.

A screening dose equivalent to ¼ IC_50_ of the viability assay was selected to investigate the inhibitory effect of HE on the colonization ability of HCT-116 and MCF-7 cell lines. The results (Table 6, Fig 3) showed that the HCT-116 cells were more sensitive to the HE than the MCF-7 cells. HCT-116 displayed no surviving colonies post-treatment, while MCF-7 showed only two definite colonies by the end of the experiment (Fig 3).

**Table 6:** Effect of *P. cattleianum* aerial parts’ *n*-hexane extract (HE) and its selective sub-fractions on the colonies’ number (CN), and survival fraction (SF from control) in colonization assay.

Tested samples	MCF-7		HCT-116	
	CN		SF		CN	SF
Control	54.0 ± 2.86	1.00 ± 0.04	81.5 ± 3.28	1.00 ± 0.04
HE	2.0 ± 0.81	0.03 ± 0.01****	0	0
Fraction I	1.0 ± 0.81	0.01 ± 0.01****	0	0
Fraction II	2.0 ± 0.81	0.03 ± 0.02****	0	0
Fraction III	NT	NT	24.5 ± 1.37	0.30 ± 0.02****
Fraction IV	24.0 ± 2.70	0.44 ± 0.05****	58.0 ± 0.49	0.71 ± 0.02****
Fraction V	NT	NT	1.0 ± 0.81	0.01 ± 0.01****

NT: not tested; No. of plated cells = 100 cells. Data presented as mean ± SEM (n = 3); values were compared using the student’s *t*-test, and the statistical significance was denoted with an asterisk as follows: ****: p < 0.0001 vs control

Concerning HE’s subfractions, HCT-116 lost the ability to form definite colonies following treatment by fractions I and II. Fraction III significantly (*P* < 0.0001) reduced colony formation efficacy (CFE) to 24%, and fraction IV displayed a significant (*P* < 0.0001), moderate inhibitory effect (58% CFE) compared to the vehicle-treated group (81% CFE). Cells treated with fraction V displayed only 1% CFE. Regarding MCF-7 cells, fractions I and II displayed significant (*P* < 0.0001) colony inhibition compared to vehicle control. Deduced from their calculated colony formation capacity, which was calculated as 1% and 2%, respectively. Lastly, fraction IV reduced CFC to 24% compared to 54% in the control vehicle group (Table 6).

#### Molecular docking of *β*-caryophyllene oxide on estrogen receptor (ER).

**Fig 4:**
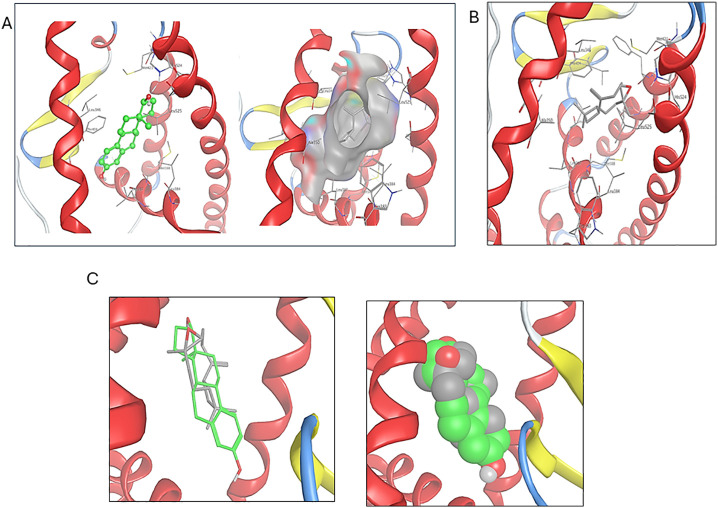
(A) DNA binding pose of estradiol at estrogen receptor ligand binding domain (PDB 1A52). (B) Predicted binding pose of *β*-caryophyllene oxide on the ER ligand binding pocket and its molecular interactions. (C) Predicted overlay of estradiol and *β*-caryophyllene oxide binding poses at the ER ligand binding pocket in stick and space-filling representation of estradiol (in green) and *β-*caryophyllene oxide (in gray) overlay.

The ER ligand-binding domain was retrieved from the Protein Data Bank (PDB) and optimized for docking experiments. As depicted in Fig 4A, the ER-ligand binding domain has two critical points of contact with its canonical ligand estradiol: a hydrogen bond contact at one pole of the pocket that accommodates the steroidal ring-D and hydrophobic van der Waals interactions along the hydrocarbon scaffold of the steroid nucleus. The chemical structure of *β*-caryophyllene oxide was energetically minimized and prepared for docking iterations at the ER ligand binding domain (ERLBD). Docking results revealed that *β*-caryophyllene oxide occupied the center of the binding pocket created by the backbone and side chains of helices 3 and 10/11 (Fig 4B), with multiple hydrophobic interactions. By comparing the virtual binding poses of *β*-caryophyllene oxide and estradiol, both molecules are nearly overlaid. On top of that, *β*-caryophyllene oxide is superposed with rings B-D of the steroid hormone (Fig 4C).

## Discussion

*Psidium cattleianum* Sabine (Family Myrtaceae) is an elite guava species native to Brazil and other tropical countries. The shrub is prized for its assorted bioactive metabolites, traditional uses, and pharmacological activities, highlighting its therapeutic significance in many aspects [4–8,31,32]. As part of our ongoing research to discover anticancer hits from *P. cattleianum,* herein, the HE has been investigated for the first time, chemically and biologically, using various chromatographic techniques and anticancer platforms. The chromatographic analysis of HE using GC-MS revealed that it is rich in a panel of metabolites, some of which have been previously noticed for the genus. For instance, cubebene, alloaromadendrene, *β*-copaene, *α*-bergamotene, *γ*-elemene, muurolene, germacrene D, and caryophyllene oxide have been previously reported in the essential oil (EO) of *P. cattleianum* [7]. On top of that the isolation of caryophyllene oxide has been described earlier from the EO of *P. cattleianum* by Oh et al. [33]*.* However, tocopherol and squalene have been reported in the leaves’ extract [34]. Likewise, *β*-sitosterol and *α/β*amyrins have been previously isolated from *P. guajava* leaves’ extract, while bisabolol, cadinol, and phytol have been identified in the leaves’ EO [35]. Although these compounds have been detected earlier, this is the first report of such a unique blend of chief constituents in high concentrations. For instance, caryophyllene oxide constitutes 12.07% of the HE of the aerial parts, while 3.39–4.81% of the leaves and flowers’ essential oil [7]. Humulenol-II was identified in our extract at 4.06% while it is reported as 1.5% in the EO of the leaves [7]. Likewise, *α*-humulene has been detected in our extract (7.42%); however, previous reports showed its detection in higher percentages (10–15%) [7]. These variations may be due to several factors, including applied temperature, extraction solvent, environmental conditions, and ecological factors. It is reported that the gradual increase in extraction temperature directly causes an exponential escalation in the percentage of extracted components. However, exceeding certain limits of heating may lead to thermal decomposition [36]. Regarding the extraction solvent, *n*-hexane is a nonpolar solvent ideal for extracting hydrophobic components. Additionally, it is credible for its low boiling point (68 °C) and cost-effectiveness [37]. Ultimately, environmental and ecological conditions significantly impact the physiological and biochemical responses of medicinal plants, hence their secondary metabolic machinery [38]. In all, *P. cattleianum* aerial parts’ HE, prepared as per our protocol, is considered a promising and rich source of variable chemical hits in high yield.

Consequently, the extract was fractionated into seven sub-fractions using column chromatography to simplify its complexity. Fractions I and II were oily and possessed an aromatic odour, so they were analyzed using GC-MS. On the contrary, fractions III-VII were non-oily; hence, we proceeded with their analysis using HPTLC hyphenated to mass spectrometry. We noticed that fraction I is prolific with non-oxygenated components (52.65%), which declined to 20.52% in fraction II. This is a logical sequence for metabolites’ elution from Silica gel column chromatography based on their polarity. The profile of fractions I and II was characterized by sesquiterpenes with the following skeletons: caryophyllanes, aromadendrans, germacrene, sesquithujanes, curcumanes, cadinanes, eremophilane, bisabolene, eudesmane, and elemane. These classes and their associated metabolites have been previously reported in different Psidium species, including *P. cattleianum* [7,33]. It is noteworthy that these classes are biosynthesized *via* the farnesyl phosphate pathway, one of the common biosynthetic pathways in Psidium species [33]. The remaining identified metabolites were related to the oplopane and clovane sesquiterpenes, as well as other miscellaneous classes that were reported for the first time in the genus Psidium.

HPTLC-MS was used to analyze fractions III-V, leading to the identification of eleven metabolites. Amongst caryophyllene, littordial C, *β-*tocopherol, and *trans*-calamenene were previously reported in *P. cattleianum* and *P. guajava*, while guavanoic acid, *p*-coumaroyl caffeoyl quinic acid, cholestane heptol, dihydroxy-oxo-ursenoic acid, hydroxyoctadecanoic acid, and heptacosanedione were detected in *P. cattleianum* extract for the first time. To the best of our knowledge, HPTLC was previously used to determine the phytochemical content of *P. guajava* [39,40]. However, this is the first time HPTLC has been adopted for profiling *P. cattleianum* extracts. As a state-of-the-art analytical technique, hyphenated HPTLC provides straightforward information about the metabolites in a complex sample. Additionally, its elite chromatographic separation, linked directly to MS detection, enables sensitive detection of minor components with minimal sample preparation [41]. In all, it is worth considering that extraction and environmental factors, as well as variability in the implementation of various analytical techniques, can play an essential role in the discovery of new chemical entities in *P. catellianeum*.

Given the significant global health burden of breast cancer (BC) and colorectal cancer (CRC), the HE was preliminarily screened for its inhibitory effect on the viability of MCF-7 breast adenocarcinoma and HCT-116 colorectal carcinoma cell lines. Both were selected based on the statistical global burden [13,14] and their in-house availability. According to the American National Cancer Institute (NCI), crude extracts are classified as potent cytotoxic agents and promoted for subsequent purification when their IC_50_ is less than 30 µg/mL [42,43]. At the same time, Ayoub and co-workers [44] consider concentrations up to 100 μg/mL for an extract to be effectively cytotoxic. Based on the IC_50_ values of *P. cattleianum* HE in the cytotoxicity assay, HE was classified as toxic with a preference for MCF-7 cells over HCT-116. This observation matches our previous reports on the selective cytotoxicity of *P. cattleianum’s* essential oils (EO) from the leaves on MCF-7 (IC_50_=5.32 μg/mL). However, the EO was 11-fold less toxic to normal WI38 cells [7]. Additionally, Mahrous et al. reported the potent cytotoxic effect of terpenoids isolated from *P. cattleianum* leaves on a panel of cancer cell lines (IC_50_=22.0-64.4 μm) with no cytotoxicity to normal human melanocytes (HFB4 cells) [45]. Accordingly, *P. cattleianum* HE is categorized as a selective, potent cytotoxic agent and is promoted as a promising source for the discovery of novel anticancer hits.

According to our characterized chemical profile, HE is fertile in diverse cytotoxic chemical scaffolds. For instance, *β*-caryophyllene oxide is renowned for its significant anticancer activities. It suppresses proliferation, induces apoptosis, and reduces angiogenesis and metastasis in various cancer cells through multiple pathways. Such as mitogen-activated protein kinase (MAPK), PI3K/mTOR, and STAT3 [46]. Another potential anticancer hit is humulene, a monocyclic sesquiterpene. Humulene is known for its apoptotic effect on CRC cells *via* targeting the death receptor 5 and caspase-8 and -3-dependent signaling pathways [47]. Last but not least, several researchers have endorsed the anticancer effects of amyrins. Reportedly, they were capable of inducing apoptosis in liver cancer cells and inhibiting the migration and colonization of breast cancer cells [48]. In addition to the major bioactive hits, we cannot neglect the additive or synergistic cytotoxic impact of the minor hits. Such as nerolidol, bisabolol, and germacrene D. For instance, da Silva and the research team reported the cytotoxic effect of germacrene D on colon, breast, and leukemia cancer cell lines [49]. Rigo and Vinante documented that bisabolol is an effective cytotoxic agent against various human cancer cells *in vitro* and *in vivo* [50]. Ultimately, Chan et al [51] have mentioned the strong cytotoxic effect of nerolidol against BT-20 breast carcinoma and HeLa cells, in addition to its potential in augmenting the effect of doxorubicin on CaCo-2 colon cancer cells [51].

Fractionation of the HE untangled its complexity and helped track potential bioactive hits. For instance, fraction I showed remarkable similarity to HE in the major cytotoxic hits such as caryophyllene oxide, humulene, and calamenene. Calamenene-type sesquiterpenoids have been noticed for their antiproliferative effect on the A2780 ovarian cancer cell line [52]. On the other side, fraction II showed similarity to HE in the presence of caryophyllene oxide, while it differed in possessing other cytotoxic sesquiterpenes such as cadalene. Reportedly, cadalene exhibited cytotoxic and anticlonogenic properties in MCF-7 and MDA-MB-468 breast cancer cell lines [53]. In all, we rationalize the enhanced activity of fraction I and, to a lesser extent, fraction II, to the dedication of chromatographic fractionation. This is because chemical scaffolds with similar chemical and, hence, biological properties have been eluted concurrently in the same fractions. Moreover, other bioactive metabolites that were previously masked by the compact nature of the HE have been revealed through the chromatographic purification process. Hence, the fraction becomes enriched with potent anticancer hits, while other inactive components have been kept to the bare minimum or eluted in later fractions.

Metastasis is the foremost cause of more than 90% of cancer fatalities [54]. It is a multi-step cascade encompassing local tumor cell migration, invasion, intravasation, exit from circulation, and colonization at distal locations [55]. At the earliest stage, treatments that target cancer cell dissemination and migration have significant potential. However, at advanced stages, aiming for cancer cell colonization becomes relevant and of great importance for effective anti-metastatic therapy. Accordingly, HE and its active fractions were investigated for their inhibitory effect on the migration and colonization of MCF-7 and HCT-116 cancer cells using wound healing and colony formation assays, respectively. The wound healing assay is a customary *in vitro* procedure for probing cancer cell migration at a two-dimensional level [56]*.* In this experiment, a cell-free area was created in the confluent monolayer. Cancer cells were spontaneously induced to move into the gap while the applied treatment was tested for its ability to inhibit this directed and coordinated movement [56]. Two screening doses equivalent to ¼ IC_50_ and ½ IC_50_ in the viability assay were used to ensure that the effect is a factual motility inhibition, not cytotoxicity [57]. Regarding colony formation inhibition, the clonogenic assay tested HE and its selected subfractions for their ability to confine the proliferation and colonization of single cancer cells. The assay can be applied to most of the adherent mammalian cells in culture. Each single surviving cell will divide and undergo a minimum of 5–6 doublings to give rise to colonies containing at least 50 cells [19]. This enables the quantification of cell survival/cell death, as well as the detection of cell proliferation and growth, based on reduced or increased colony size [58].

Emerging verifications have defined the complex poly-antineoplastic profile of caryophyllane sesquiterpenes. Examples are, but are not limited to, *β*-caryophyllene, *β*-caryophyllene oxide, and *α*-humulene, which constitute the major identified compounds in the active fractions I and II. Caryophyllane sesquiterpenes suppress, chemo-sensitize, and modulate various intracellular cascades. Thus, they affect cancer proliferation, migration, invasion, colonization, and sensitivity to chemotherapy in different cancer cell lines, including MCF-7 [59]. Dahham and co-workers reported that extracts rich in *β*-caryophyllene suppress cancer cell motility, invasion, and colony formation [60]. The antiproliferative and antimigratory effects of *β*-caryophyllene have been revealed through a mechanistic study using human umbilical vein endothelial cells (HUVECs). However, the notable anti-tumorigenic activity of *β*-caryophyllene has been observed on HCT-116 human colorectal tumor xenograft models (both ectopic and orthotopic) through targeting VEGF [61]. On the other side, Park et al [62] have reported the ability of caryophyllene oxide to inhibit the intrinsic activation of the PI3K/mTOR downstream signaling pathway. Hence, it downregulated the expression of genes implicated in cell proliferation, metastasis, and angiogenesis [62]. Aside from the anticipated bioactivity of fractions I and II, we observed that some fractions, such as fraction IV, exhibited stronger cytotoxicity and better inhibition of colonization than the HE, which was contrary to the results obtained from the viability inhibition assay. In fact, fraction IV comprises two bioactive metabolites, namely, littordial C and caryophyllene. Littordial C is the formyl-phloroglucinol derivative of *β*-caryophyllene. Previous reports documented its significant cytotoxicity against MDA-MB-231 (breast cancer cells) and B16 (melanoma cells) [29]. whilst the anticancer potential of caryophyllene is well-documented across a panel of various cell lines [61,63,64]. The potency of Fr IV was prominent in the CFE as the assay is non-colorimetric; hence, there is no interference between the tested sample and the readout or reagents used, which is frequently seen with the MTT colorimetric assay. So, it is considered an accurate and sensitive assay. Additionally, unlike most cytotoxicity assays with an exposure time of less than 48 h, the CFE assay can be regarded as a sub-chronic assay as the exposure time lasts for several days; hence, it reflects true viability and the capacity of cells to proliferate. Thus, in the CFE assay, direct toxic effects on each cell are determined [58]. To sum up, *P. cattleianum* HE and its bioactive fractions I, II, and IV contain bioactive metabolites that effectively inhibit the migration and colonization of metastatic cancer cells.

To predict the macromolecular interaction entity that mediates the anticancer activity, we implemented the molecular modeling platform. In this context, *β*-caryophyllene oxide (the major metabolite identified in the HE and its active fractions) was selected and tested *in silico* to establish an energetically favored complex with estrogen receptor (ER). ER-mediated oncogenic phenotypes of ER-positive breast and colon cancer cells are well established in the literature. For instance, ER overexpression is correlated with poor prognosis and overall survival in colorectal cancer patients, which is documented to have a significant interplay with oncogenic KRAS [65]. Additionally, the crucial role of hormone receptor signaling pathways in tumorigenesis and the therapeutic response of breast cancer is well studied. The ER ligand binding domain (LBD) has two critical points of contact with its canonical ligand estradiol: a hydrogen bond and hydrophobic van der Waals. The 17*β*-hydroxy group on the steroidal ring-D of estradiol accepts a hydrogen bond from the NH imidazole side chain of His524. Additionally, the hydrophobic propyl side chain of Leu387 binds to ring-A of the steroid hormone *via* a hydrogen-arene interaction. Furthermore, ring A is also embedded in a highly hydrophobic receptor pocket stuffed with non-polar amino acid side chains, including Met388, Leu384, Leu391, and Phe404. Based on the observations, it was concluded that hydrophobic interactions/forces could significantly contribute to the binding of ER modulators.

At a glance, *β*-caryophyllene oxide has a hydrophobic scaffold, consisting of the bicyclo [7.2.0] undec-4-ene framework. Thus, we proposed that the major driving force of *β*-caryophyllene oxide-ER interactions would be the hydrophobic and van der Waals interactions. To test this hypothesis, the chemical structure of *β*-caryophyllene oxide was energetically minimized and prepared for docking iterations at the ER ligand binding domain (ERLBD). Examining the predicted binding poses of *β*-caryophyllene oxide revealed that they possessed multiple spots of hydrophobic interactions with ER binding groove. Where the *gem*-dimethyl cyclobutane is projected towards a small and shallow sub-pocket created by the hydrophobic side chains Ala350 and Leu387. The 9-membered cyclononene moiety is positioned at the core of the binding pocket through multiple hydrophobic interactions with side chains of Leu346, Phe404, Met421, and Leu525. This indicates that the majority of the active site’s amino acids experience hydrophobic interactions with the carbocyclic framework of *β*-caryophyllene oxide. Additionally, we observed that the epoxide ring is directed to the imidazole side chain of His524 (a conserved amino acid), which consolidates the binding to the 17*β*-OH group of estradiol through a hydrogen bonding interaction. We also measured the molecular distance between the oxygen atom of *β*-caryophyllene oxide and the imidazole NH of His524 (4.6 Å), and we found that it was sufficient to create an energetically meaningful hydrogen bonding interaction. Nevertheless, we speculated that a water bridge can exist to perform an energetically favored hydrogen bonding interaction between the epoxide and His524 NH side chain. To sum up, the docking of *β*-caryophyllene oxide at the ER ligand binding domain revealed an energetically favorable binding pose driven through hydrophobic interactions. The binding of *β*-caryophyllene oxide to ER could rationalize, at least in part, the mechanistic pathway of its anticancer effect on ER-positive breast and colorectal cancer cells.

## Conclusion

The *n*-hexane extract (HE) of *Psidium cattleianum* Sabine aerial parts demonstrated a rich chemical profile, with a notable abundance of bioactive metabolites. GC-MS profiling of the extract identified at least thirty-two compounds, among which *β*-caryophyllene oxide (12.07%), humulene (7.42%), *α-*amyrin (6.31%), and tocopherol (7.34%). GC-MS and HPTLC-MS analysis of HE’s subfractions revealed a variety of bioactive molecules, including hydrocarbons, fatty acids, and terpenoids. Key detected metabolites include globulol, cadalene, *β*-caryophyllene oxide, littordial C, guavanoic acid, and *β*-tocopherol. Even though GC-MS and HPTLC-MS are efficient, sensitive, and reliable tools for metabolite identification, setting quality control parameters for both experiments is considered a study limitation. From the bioactivity perspective, HE exhibited significant *in vitro* anticancer properties by suppressing cell viability, migration, and colony formation in HCT-116 and MCF-7 cancer cell lines at low μg/mL concentrations. Importantly, subfractions enriched in caryophyllene oxide showed enhanced anticancer activity, highlighting the possible synergistic effects of caryophyllene oxide with other bioactive compounds. Molecular docking supported the mechanistic role of *β-*caryophyllene oxide as an estrogen receptor (ER) modulator in ER-dependent BC and CRC types. Caryophyllene oxide displayed a favorable binding pose *via* hydrophobic van der Waals forces, hydrogen bonding, and perfect ring alignment. In conclusion, these findings support the potential of *P. cattleianum n*-hexane extract and its active subfractions as promising complementary products for breast and colon cancer management. Future research is highly warranted to validate these findings in a relevant preclinical model.

## Supporting information

S1 Fig Total ion chromatograms (TIC) obtained from Gas chromatography-Mass spectrometry (GC-MS) analysis of the *n*-hexane extract (HE) of *P. cattleianum* aerial parts. The positive and negative ESI-MS spectrum of the identified metabolites from fractions II, IV, and V.
(DOCX)
