# Supplementary material for: Phytochemical characterization and anticancer potential of Psidium cattleianum Sabine aerial parts’ n-hexane extract and its subfractions
Source: PLoS One. 2025 Oct 27;20(10):e0335134. doi: 10.1371/journal.pone.0335134 (PMC12558511; doi:10.1371/journal.pone.0335134)
Supplement: S1 Fig — The positive and negative ESI-MS spectrum of the identified metabolites from fractions II, IV, and V. (DOCX) [file pone.0335134.s001.docx]

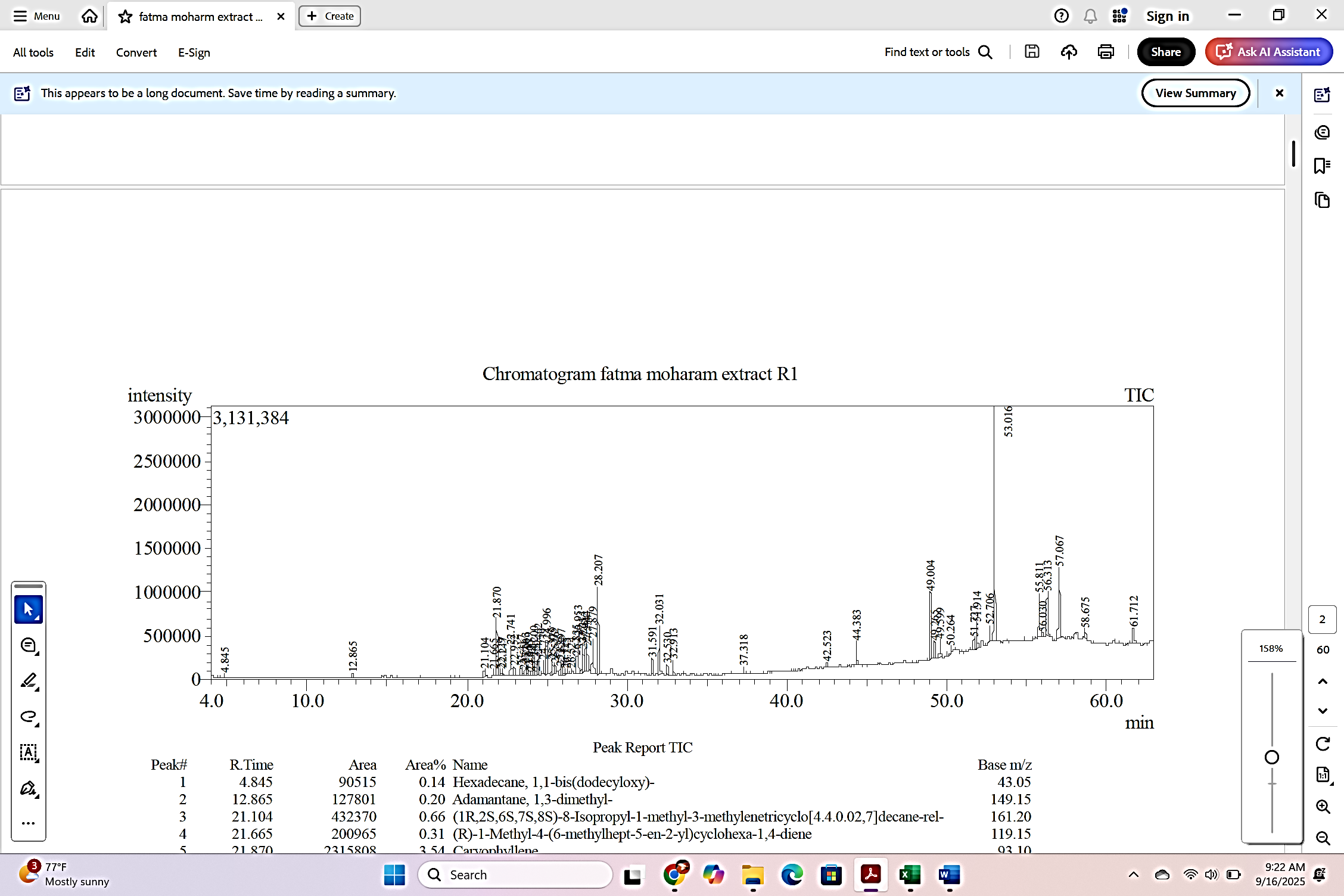


Total ion chromatogram (TIC) obtained from Gas chromatography-Mass spectrometry (GC-MS) analysis of the n-hexane extract (HE) of P. cattleianum aerial parts


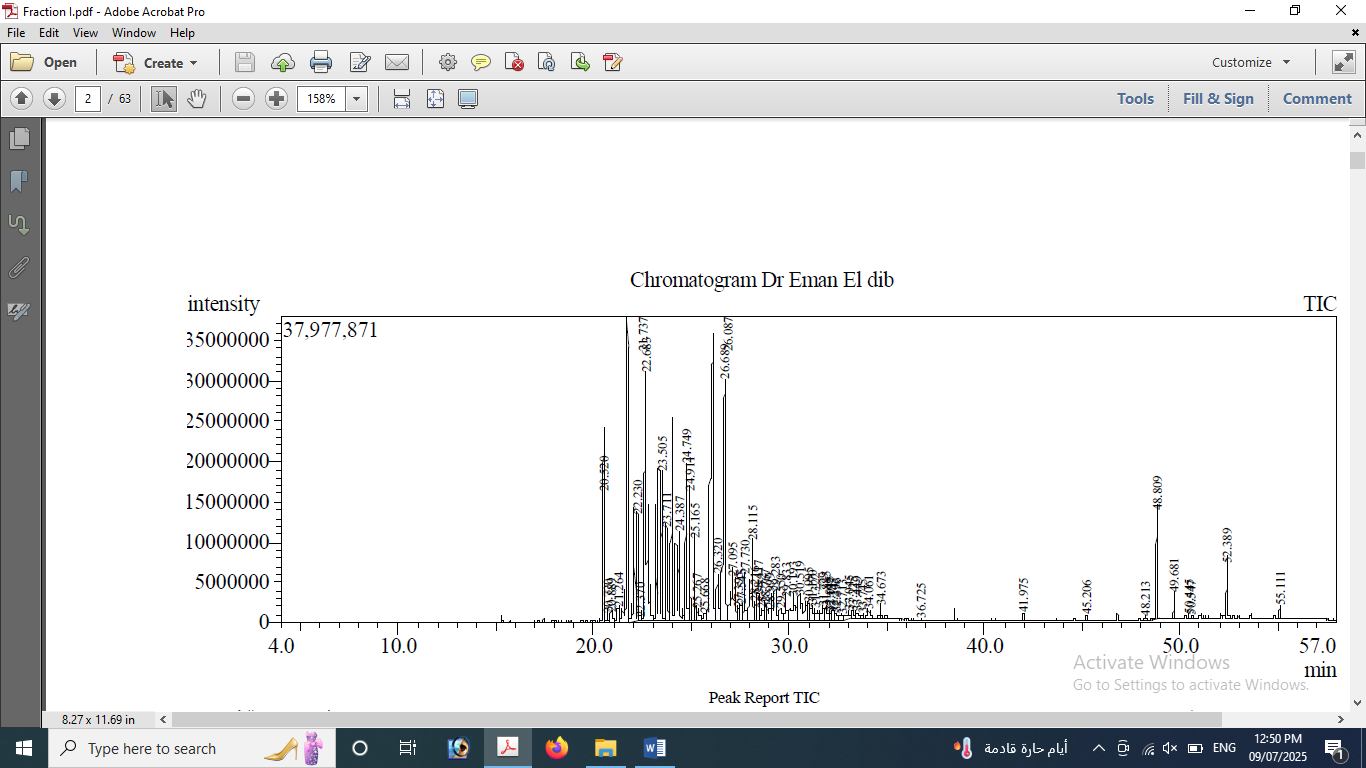
Total ion chromatogram (TIC) obtained from Gas chromatography-Mass spectrometry (GC-MS) analysis of fraction I from *P. cattelianum* aerial parts’ *n*-hexane extract


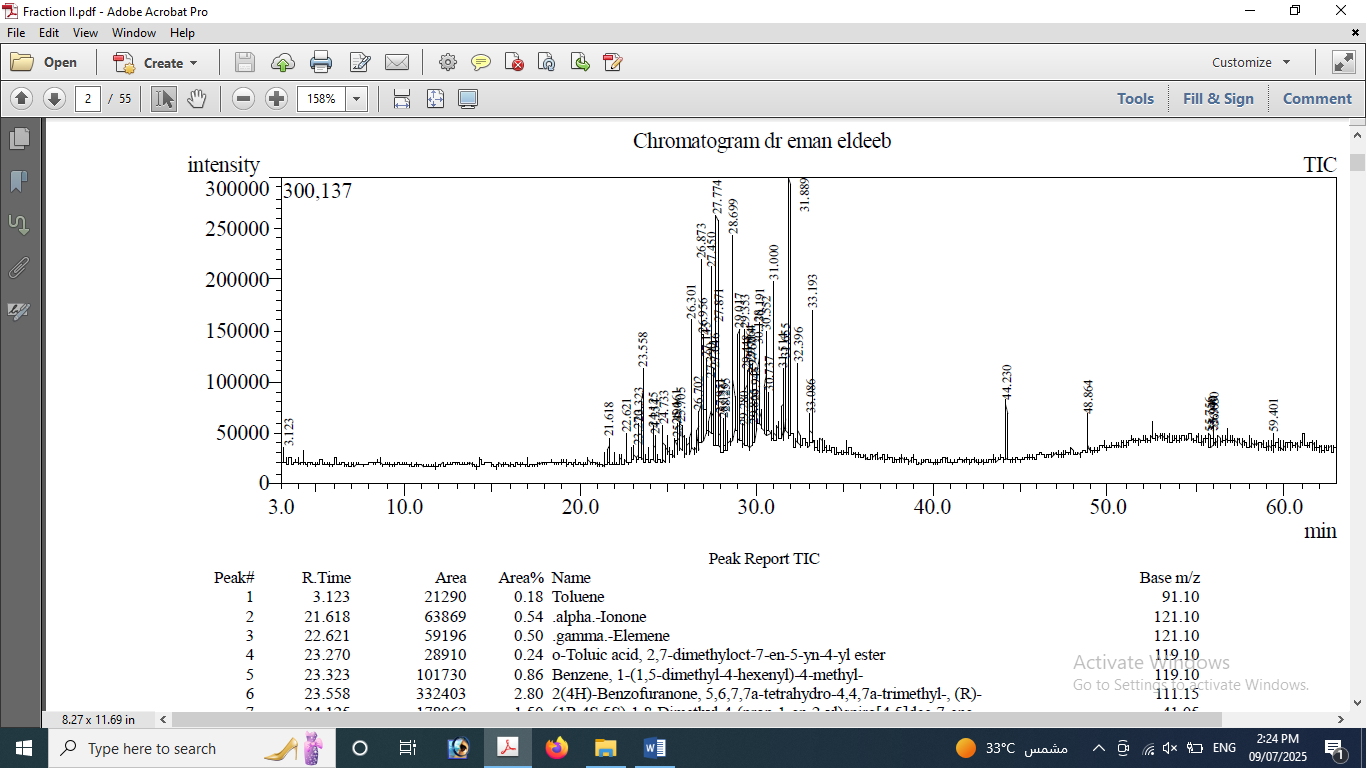


Total ion chromatogram (TIC) obtained from Gas chromatography-Mass spectrometry (GC-MS) analysis of fraction II from *P. cattleianum* aerial parts’ *n*-hexane extract


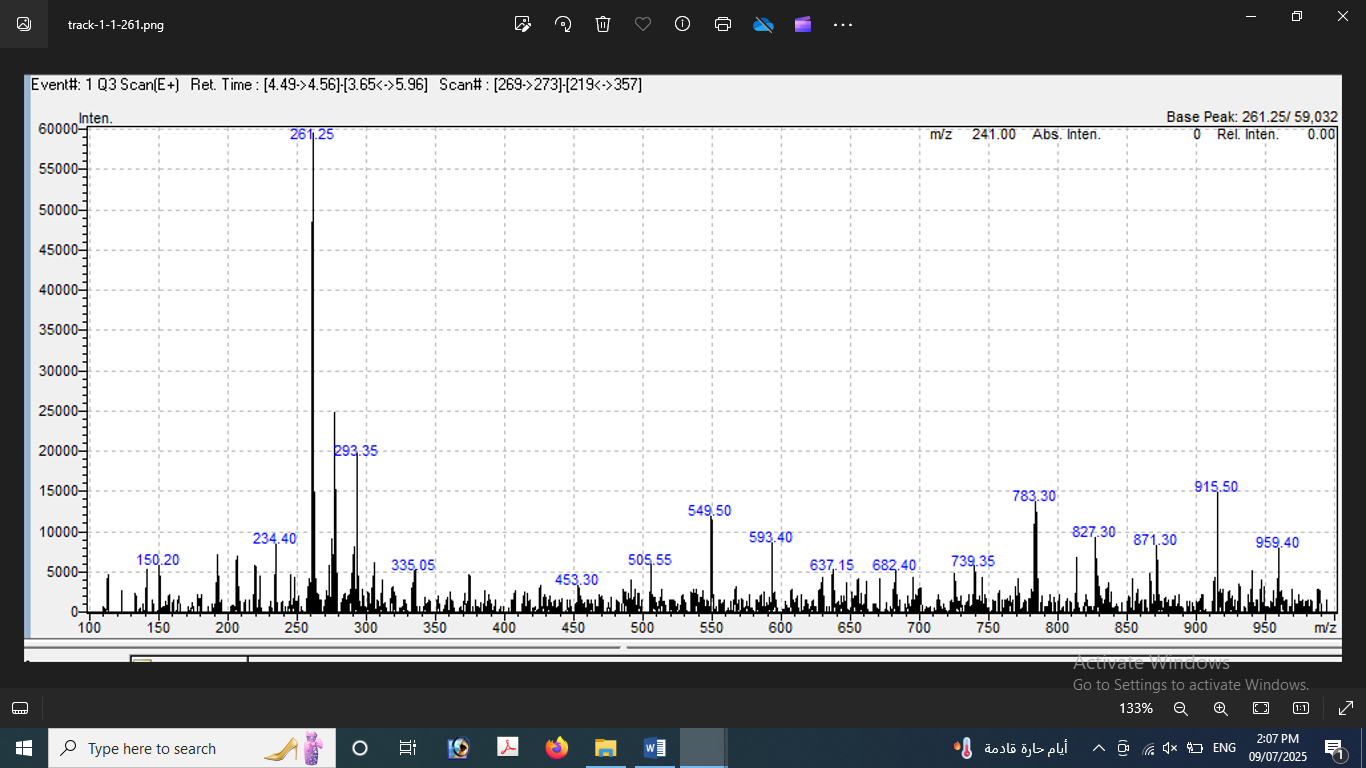


Positive ESI-MS spectrum of nonadecatetraene


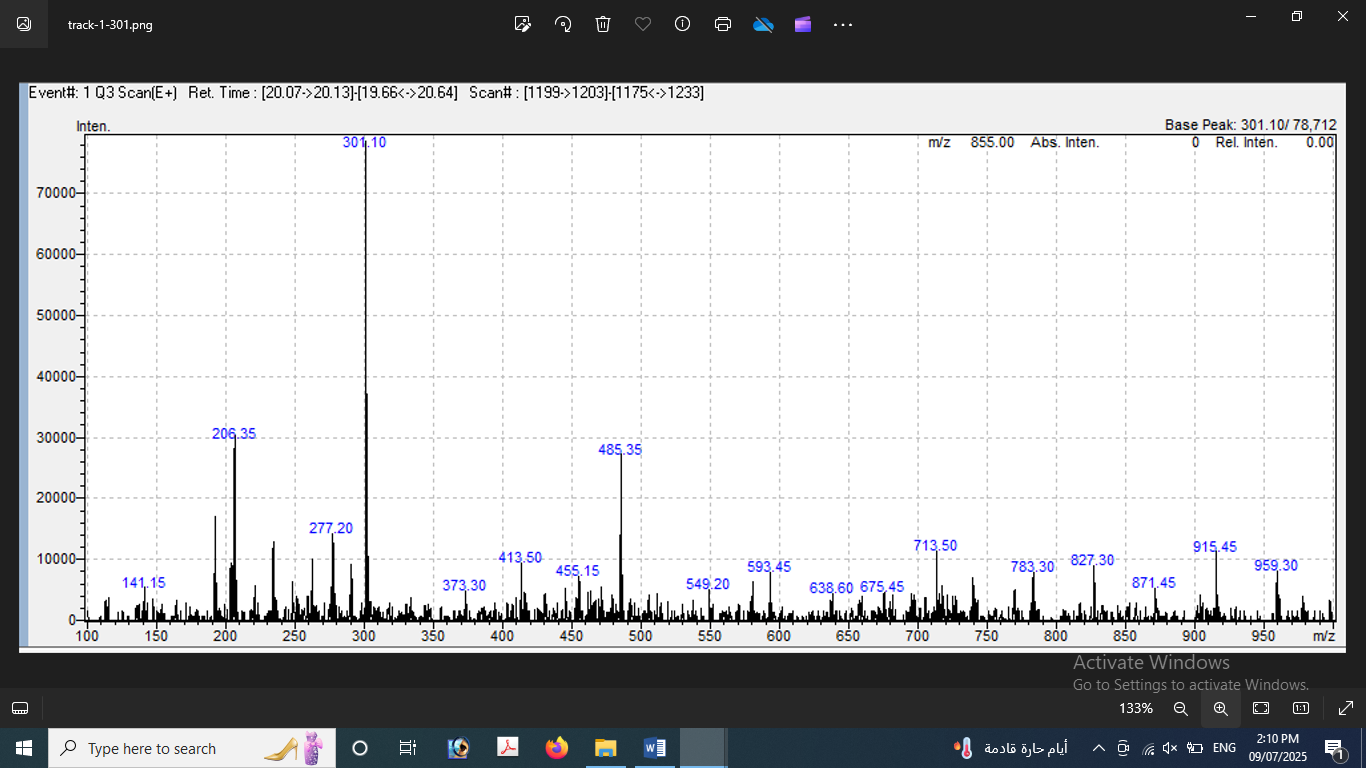


Positive ESI -MS spectrum of hydroxyoctadecanoic acid


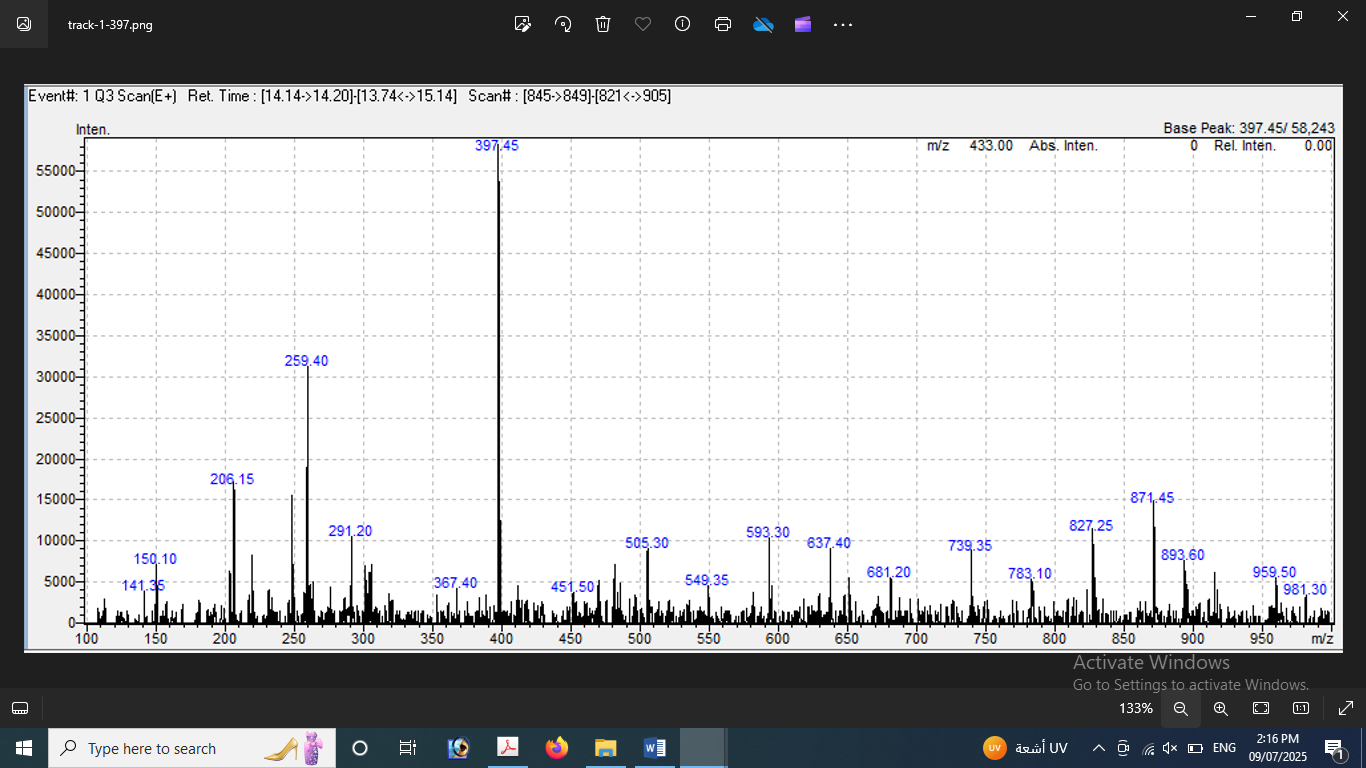


Positive ESI-MS spectrum of heptacosanol


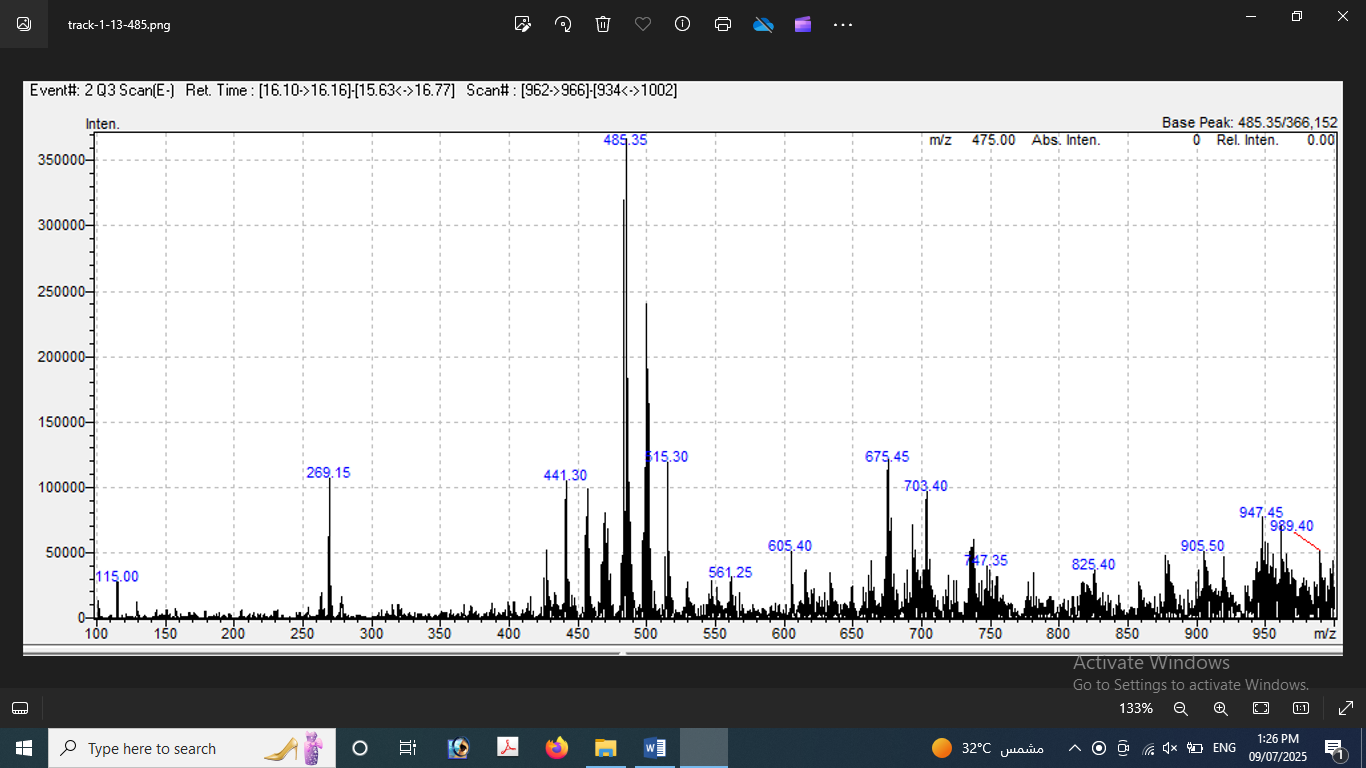


Negative ESI-MS spectrum of dihydroxy-oxo-ursen-oic acid


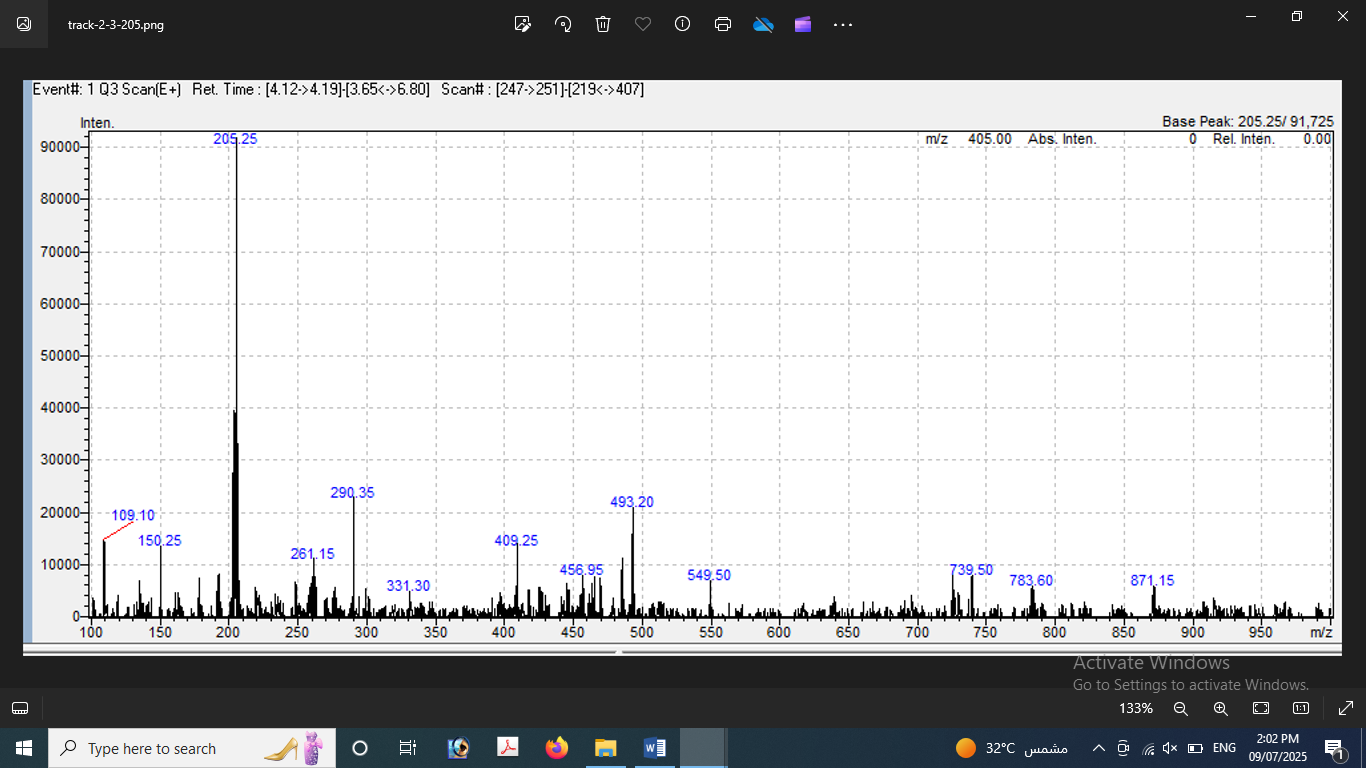


Positive ESI-MS spectrum of caryophyllene


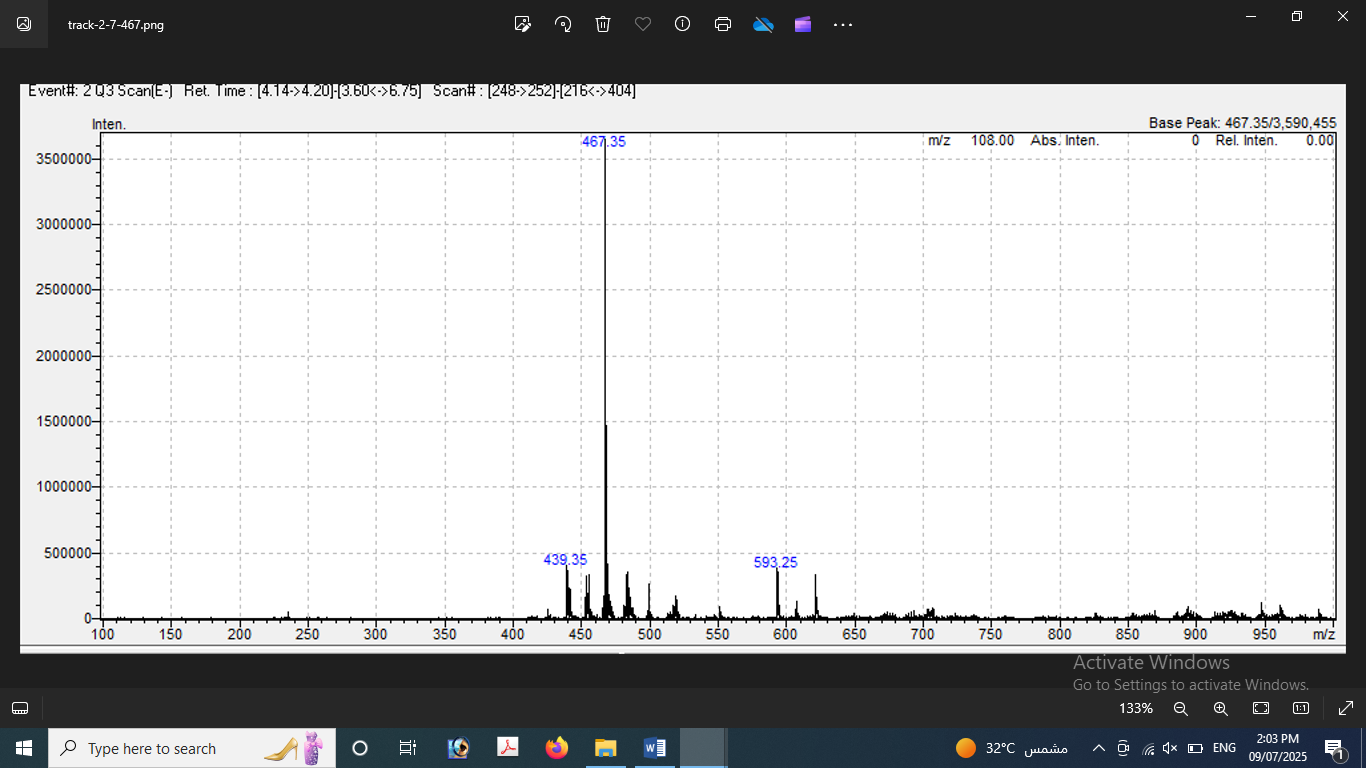


Negative ESI-MS spectrum of littordial C


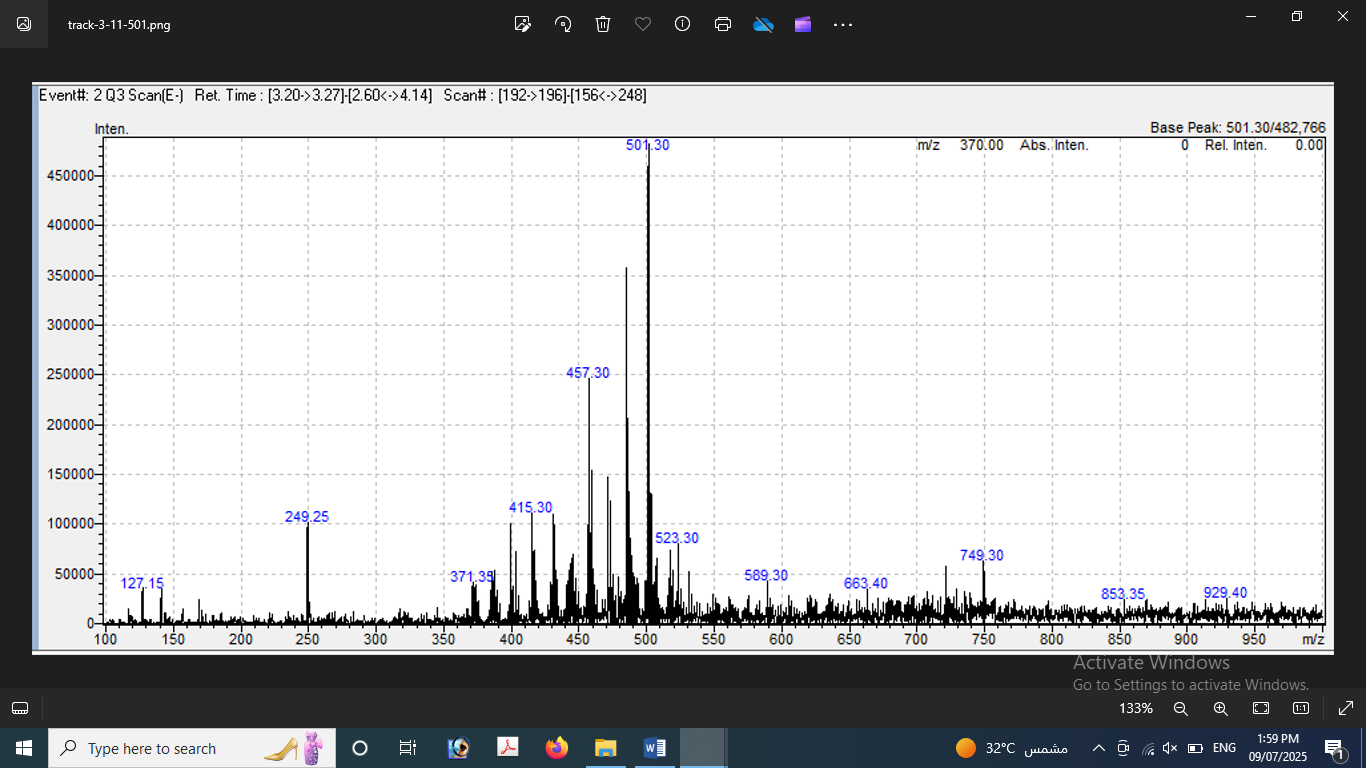


Negative ESI-MS spectrum of guavanoic acid


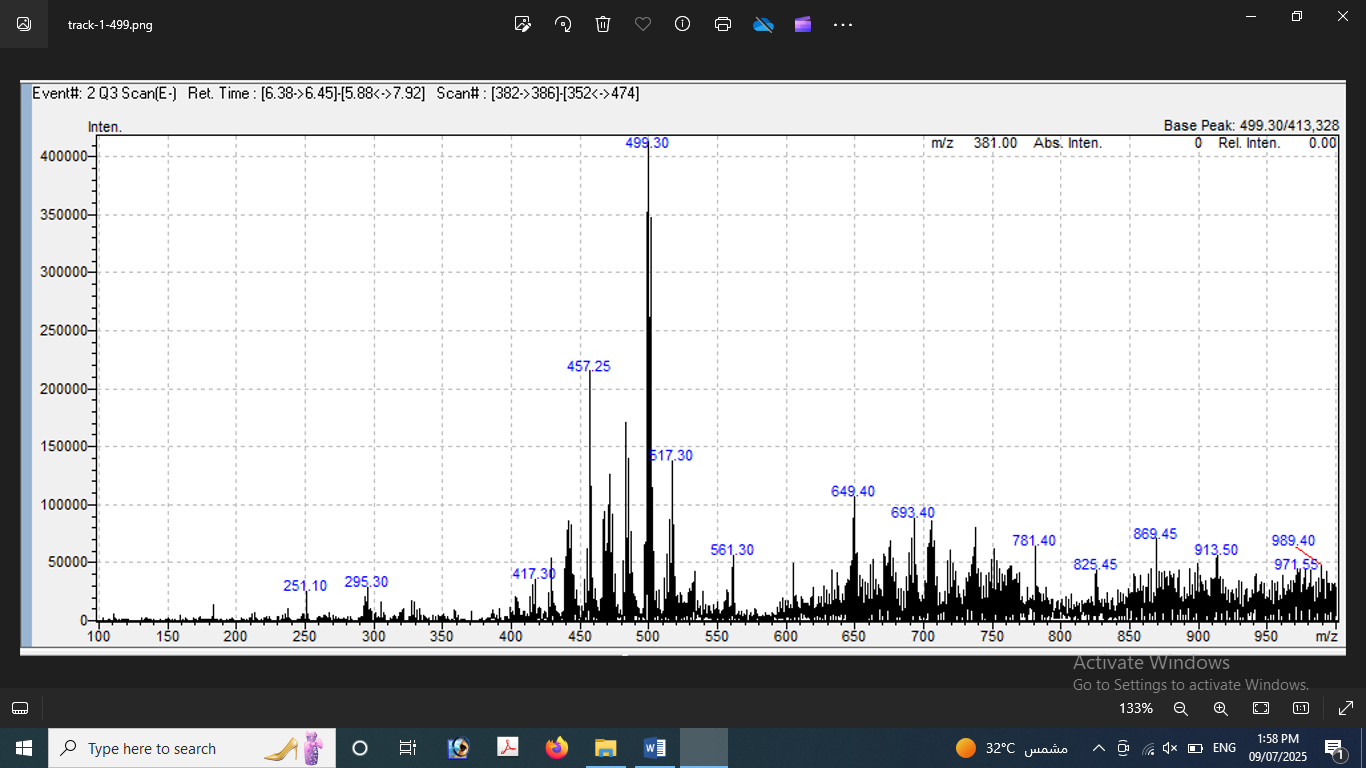


Negative ESI-MS spectrum of *p*- coumaroylcaffeoylquinic acid


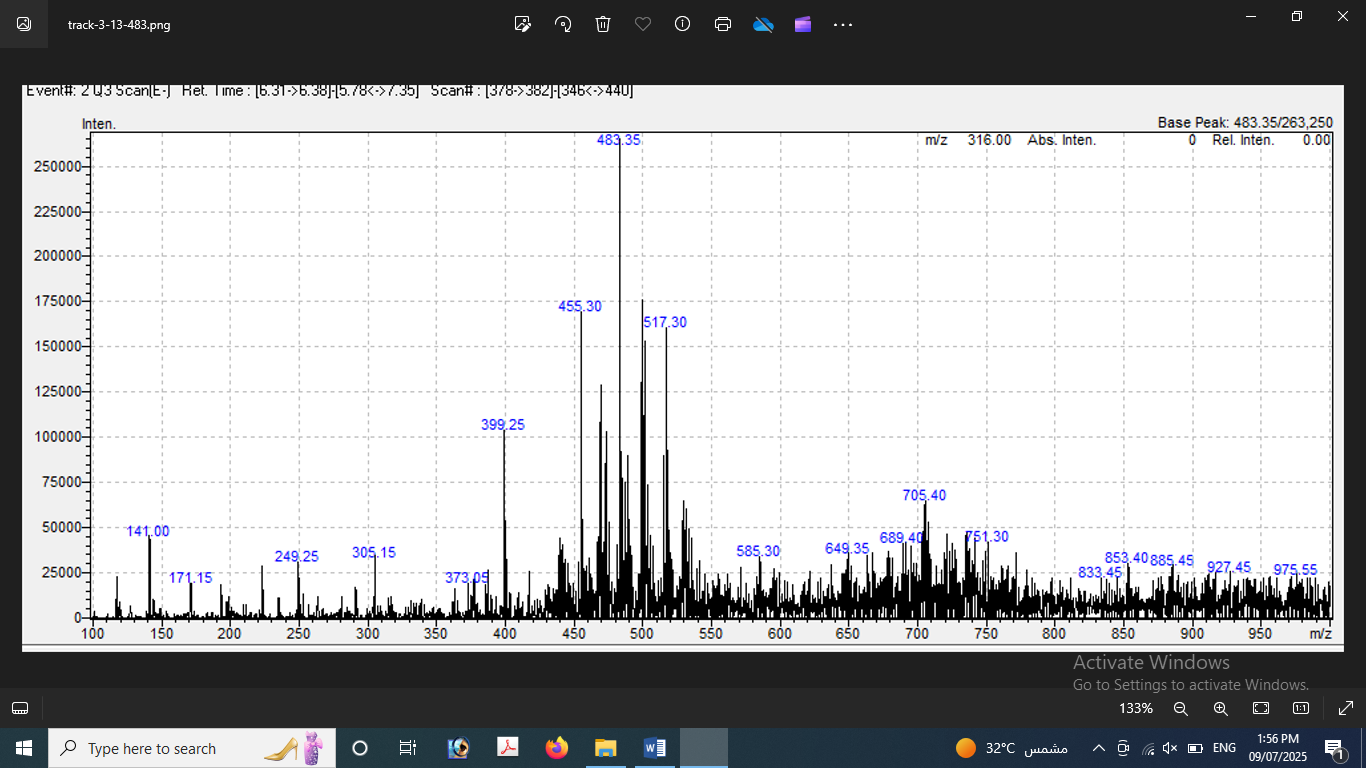


Negative ESI-MS spectrum of cholestane heptol


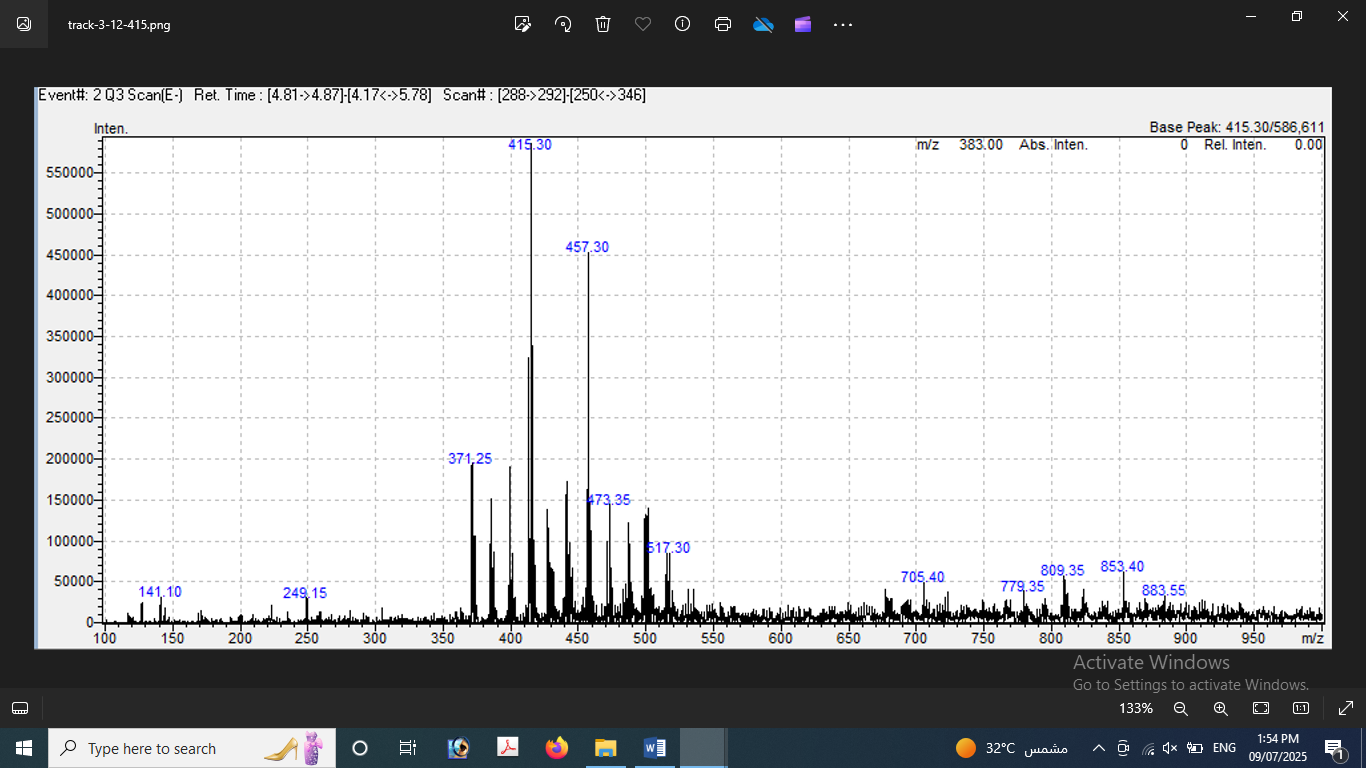


Negative ESI-MS spectrum of *β*-tochopherol


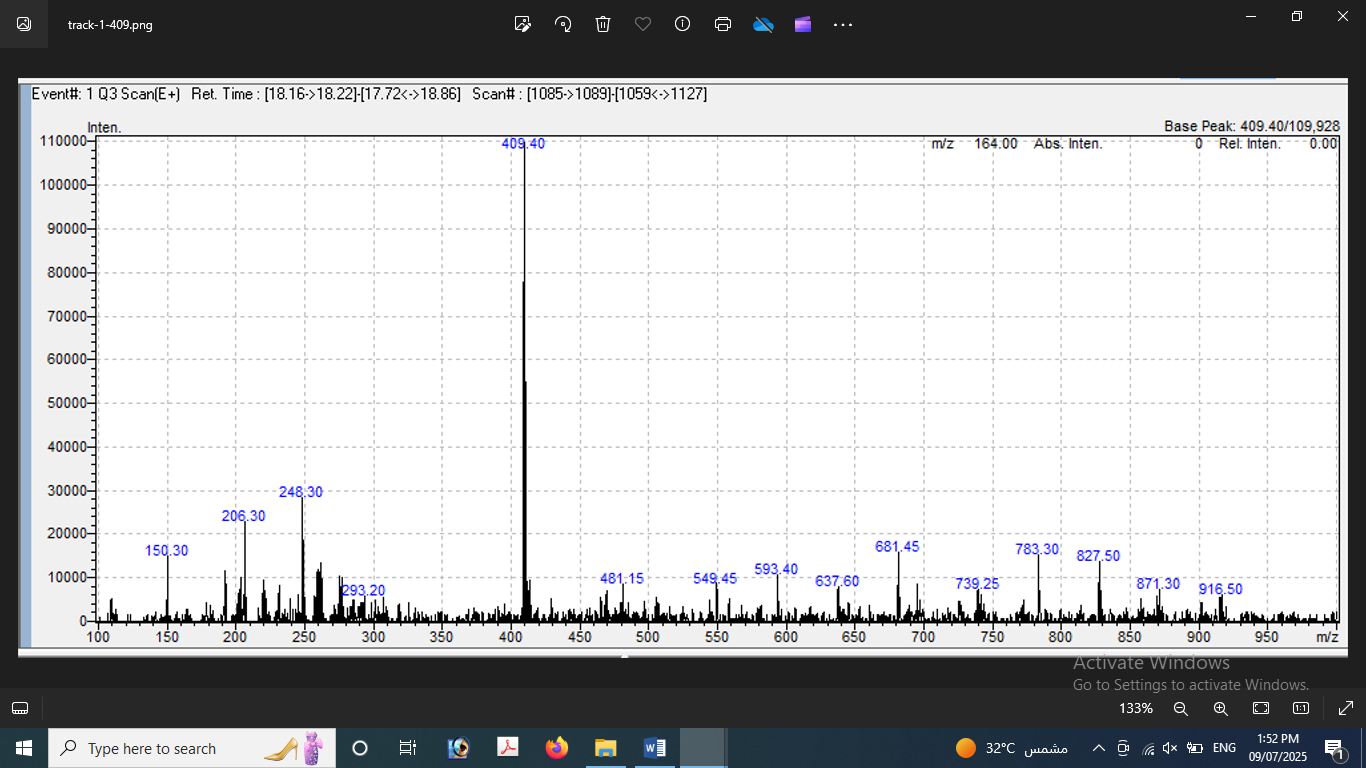


Positive ESI-MS spectrum of heptacosanedione


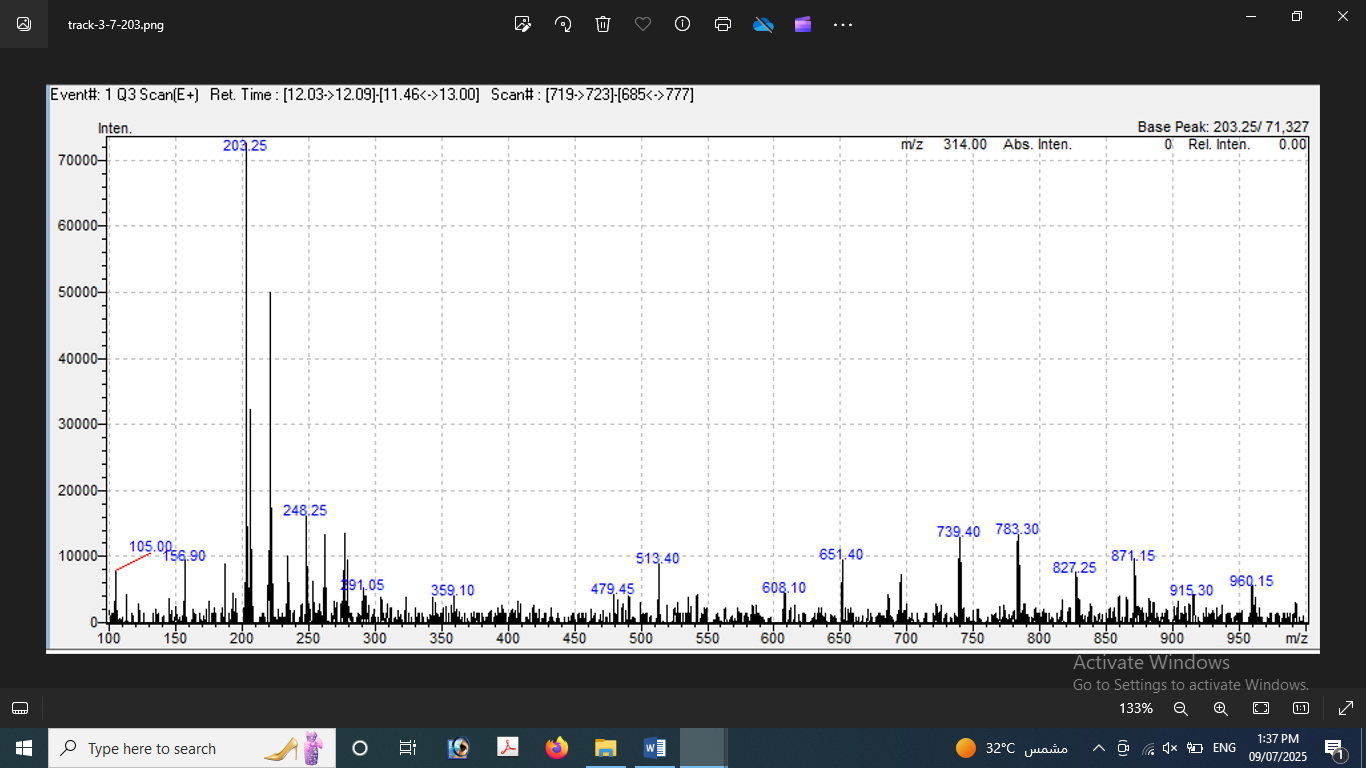


Fig. Positive ESI-MS spectrum of *trans*-calamenene


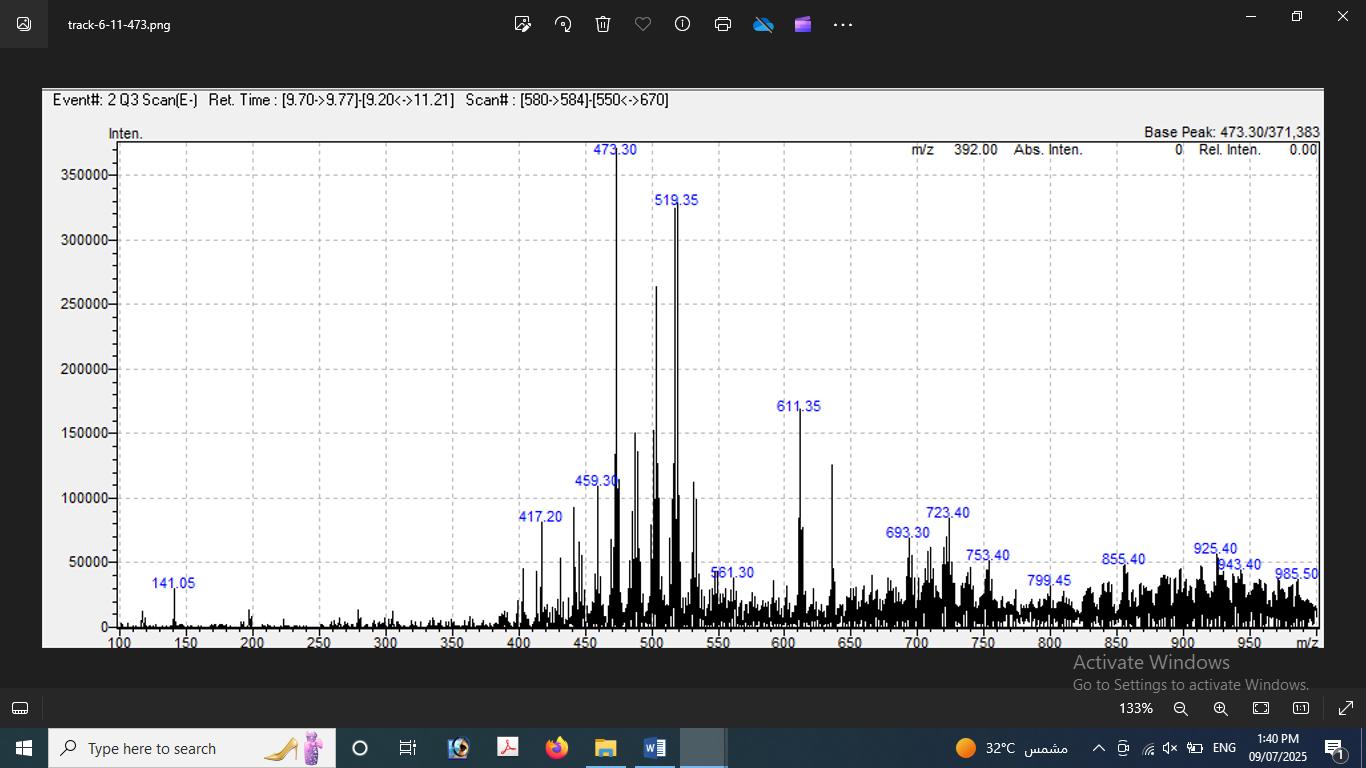


Negative ESI-MS spectrum of guapsidial A
